# Quorum sensing and iron-dependent coordinated control of autoinducer-2 production via small RNA RyhB in *Vibrio vulnificus*

**DOI:** 10.1038/s41598-021-04757-9

**Published:** 2022-01-17

**Authors:** Keun-Woo Lee, Yancheng Wen, Na-Young Park, Kun-Soo Kim

**Affiliations:** 1 Department of Life Sciences, Sogang University, Baekbeom-Ro, Mapo-Gu, Seoul, 121-742 Korea; 2 Present Address: Key Laboratory of Ministry of Education for Gastrointestinal Cancer Research Center for Molecular Medicine, Fujian Medical University, Fuzhou, Fujian People’s Republic of China

**Keywords:** Microbiology, Molecular biology

## Abstract

Roles for the non-coding small RNA RyhB in quorum-sensing and iron-dependent gene modulation in the human pathogen *V. vulnificus* were assessed in this study. Both the quorum sensing master regulator SmcR and the Fur-iron complex were observed to bind to the region upstream of the non-coding small RNA RyhB gene to repress expression, which suggests that RyhB is associated with both quorum-sensing and iron-dependent signaling in this pathogen. We found that expression of LuxS, which is responsible for the biosynthesis of autoinducer-2 (AI-2), was higher in wild type than in a *ryhB*-deletion isotype. RyhB binds directly to the 5′-UTR (untranslated region) of the *luxS* transcript to form a heteroduplex, which not only stabilizes *luxS* mRNA but also disrupts the secondary structure that normally obscures the translational start codon and thereby allows translation of LuxS to begin. The binding of RyhB to *luxS* mRNA requires the chaperone protein Hfq, which stabilizes RyhB. These results demonstrate that the small RNA RyhB is a key element associated with feedback control of AI-2 production, and that it inhibits quorum-sensing signaling in an iron-dependent manner. This study, taken together with previous studies, shows that iron availability and cell density signals are funneled to SmcR and RyhB, and that these regulators coordinate cognate signal pathways that result in the proper balance of protein expression in response to environmental conditions.

## Introduction

Bacterial pathogens are exposed to a variety of stressors either in the natural environment or in the host environment during the infection process, such as nutrient restrictions, temperature changes, osmotic stress, and oxidative stress^1^. These organisms have evolved sophisticated mechanisms to control gene expression in these diverse environments by sensing relevant environmental factors and rapidly adapting to improve both survival and pathogenicity. It is well known that iron plays an important role in regulating virulence factors in pathogenic bacteria^2^. Fur, ferric uptake regulator, is a well-studied regulatory factor that controls the expression of numerous genes in association with iron^3,4^. Similar examples are documented for *V. vulnificus*: Fur increases the expression of heme receptors in the presence of heme^5^, Fur represses genes encoding the biosynthesis and uptake of the siderophore vulnibactin^6^, and Fur suppresses quorum-sensing by modulating both the master regulator *smcR* and Qrrs, the quorum-sensing regulatory small RNAs^7,8^.

Bacterial cell density is another important factor influencing a wide range of cellular activities including the virulence of pathogenic bacteria. Regulation in response to cell density is achieved through a quorum-sensing pathway that monitors the accumulation of small diffusible signaling molecules produced by cognate cells in a closed space at high cell density, and this subsequently influences the expression of genes related to numerous functions such as survival, biofilm formation, and virulence^9–11^. The quorum-sensing pathway in *V. vulnificus* is similar to that of *Vibrio harveyi* and *Vibrio cholerae*. *V. vulnificus* harbors a homolog of *V. harveyi luxS*, which encodes an enzyme for the biosynthesis of the autoinducer-2 signaling molecule^12,13^. The genome sequence of the well-studied *V. vulnificus* strain MO6-24/O (GenBank accession number CP002469.1 for chromosome I and CP002470.1 for chromosome II)^14^ revealed that there is no biosynthetic gene for autoinducer-1 (AI-1; homoserine lactone molecule) or cholera autoinducer-1 (CAI-1). However, LuxPQ, the cognate receptor for autoinducer-2 (AI-2) and homologues of LuxU and LuxO involved in a phospho-relay in *V. harveyi*^15–19^ have been identified. The AI-2 signal converges to LuxO, a nitrogen regulatory protein (NtrC) homolog, and is transduced to the master regulator SmcR^20–22^. In addition, this pathogen harbors yet another quorum-sensing pathway that is mediated by cyclic dipeptide^23–25^.

RyhB, a non-translated small RNA, was first identified as important for iron metabolism in *E. coli*^26^. Since that time, genes homologous to RyhB have been found and characterized in the context of the iron regulon in many other bacteria such as *V. cholerae, Salmonella typhimurium*, *Yersinia pestis*, and *Shigella dysenteriae*^27–30^. Transcription is negatively regulated by Fur in the presence of iron. Under iron-limiting conditions, this repression is relieved and RyhB then represses genes encoding iron-containing proteins, such as superoxide dismutase and succinate dehydrogenase^26,31^. In addition, RyhB also activates the expression of *shiA,* a gene encoding shikimate permease, which is associated with siderophore synthesis, by directly pairing with the 5′-untranslated region of the *shiA* mRNA in *E. coli*^32^. RyhB also translationally down-regulates *fur* expression^33^. In *V. cholerae*, a *ryhB* mutant showed decreased mobility and poor biofilm formation compared to wild type^27^. RyhB represses SodB expression by pairing with the 5′-untranslated region of the *sodB* mRNA and causing the coupled degradation induced by RNaseE^34,35^. The sRNA chaperone Hfq is essential for this process as it binds and protects RyhB from RNase E degradation, thereby extending its half-life^34,36–38^.

Iron is essential for the growth of living organisms, but low solubility in the environment of a living cell makes this element a limiting factor for growth. High cell density leads to an even more severe iron stringency. Meanwhile, when intracellular iron is in excess, radicals that compromise the viability of cells are generated^39^. Therefore, it is expected that cell density and iron availability are intertwined and that these factors work together to control the expression of related genes. Here we report a new connection between quorum-sensing and the iron stress response mediated by the small non-coding RNA RyhB in *V. vulnificus*. The results of this study provide evidence for a close relationship between iron levels and cell density in terms of gene regulation and also add to the increasingly long list of roles for non-translated small RNA in pathogenic bacteria.

## Results

### Identification of the transcription start site of RyhB and evidence for Hfq-dependent stabilization of the RNA

**Figure 1:**
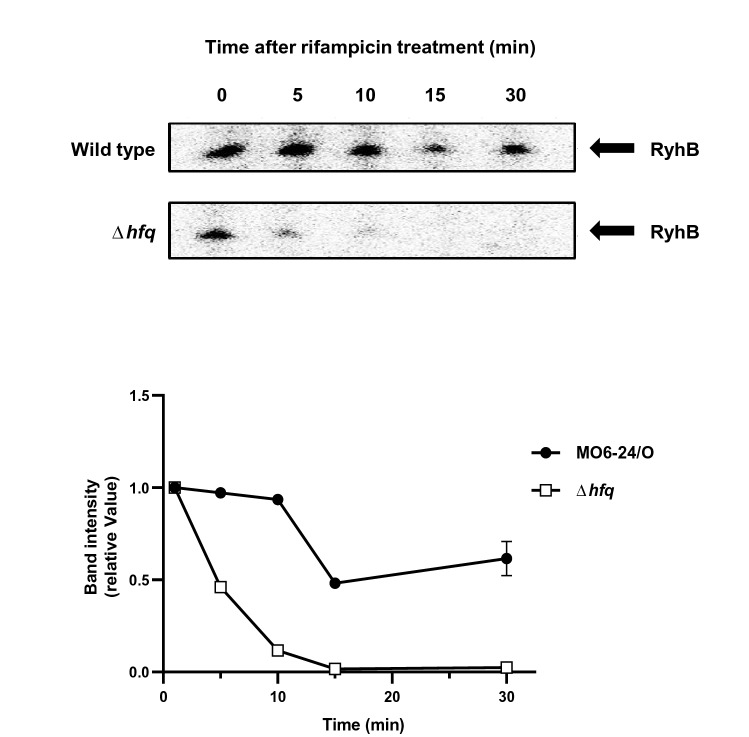
Hfq stabilizes the transcript of *ryhB*. *V. vulnificus* MO6 and *hfq* mutant (Δ*hfq*) were cultured in LB broth for 3 h, at which point 200 μM of 2, 2′-dipyridyl was added and incubation continued for another hour. Samples were then treated with 250 g/ml of rifampicin and collected at time intervals for RNA quantification. Intensities of bands were quantitatively measured using a densitometer and the results are shown as a graph in the lower panel.

Our previous study showing the repression of *smcR* by the Fur-iron complex in *V. vulnificus*^7^ led us to further investigate the relationship between quorum-sensing and iron in this pathogen. In both *E. coli* and *V. cholerae*, the Fur-iron complex represses *ryhB*, a gene encoding a small RNA^26–28^. Therefore we speculated that if any small RNAs affect quorum-sensing in an iron-dependent manner in *V. vulnificus*, the most likely candidate would be RyhB. A search of the genome sequence of *V. vulnificus* revealed a 224-bp gene with 56% similarity to the *ryhB* of *V. cholerae*. This gene mapped between the genes encoding DNA polymerase I (GenBank accession number VVMO6_02895) and porphobilinogen (VVMO6_02896). The transcription start site was identified through primer extension experiments and typical − 10 and − 35 consensus sequences also were observed to be present (Supplementary Fig. S1). The chaperone Hfq is required to stabilize RyhB in *E. coli*^35^. Therefore, we looked for a similar role in *V. vulnificus* and compared levels of the RyhB transcript in wild type and a Δ*hfq* mutant at several time points after rifampicin was added to pause transcription. As shown in Fig. 1, RyhB from wild type is still detected up to 30 min after rifampicin treatment with a half-life of about 30.7 min. On the other hand, in the *hfq* mutant RyhB was degraded quickly with a half-life of about 7.5 min, and was barely detectable at 15 min following the rifampicin treatment (Fig. 1).

### Fur represses RyhB expression in the presence of iron

**Figure 2:**
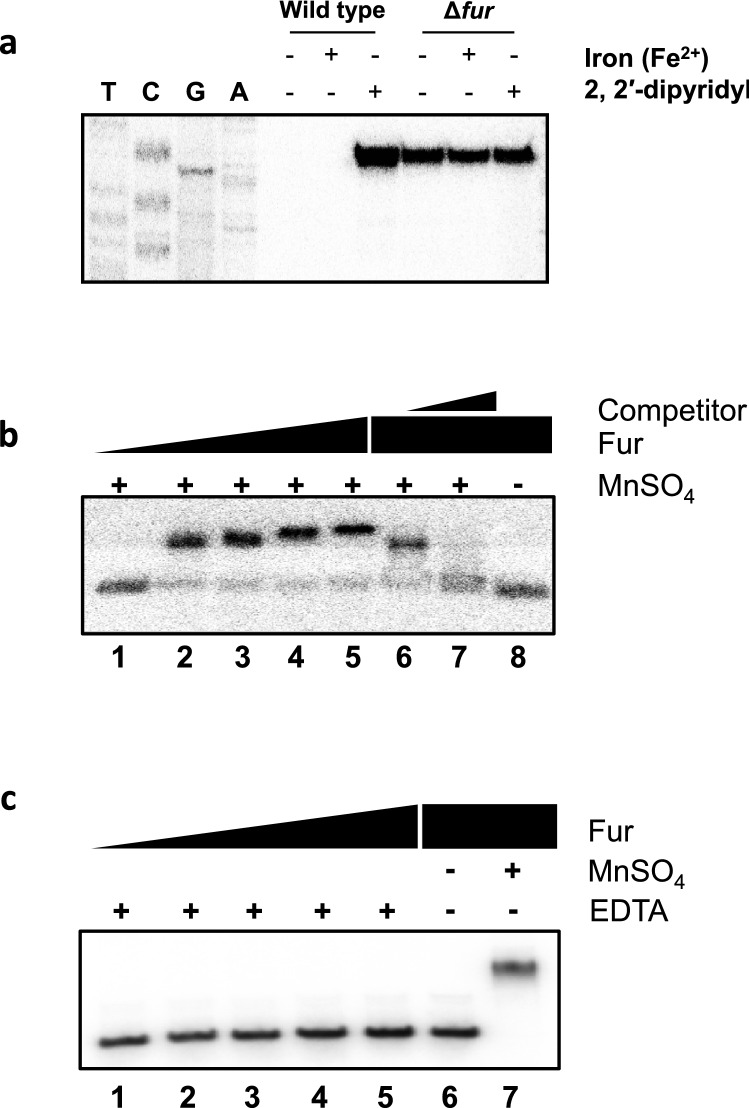
Expression of RyhB is dependent on Fur and iron concentrations. (**a**) Primer extensions were carried out to assess the level of RyhB expression with and without Fur and iron. Wild type MO6 and a *fur* mutant were cultured in LB supplemented with either 25 μM of FeSO_4_ or 200 μM 2, 2′-dipyridyl. After RNA extraction, the RyhB transcript was quantified by primer extension using primer RyhB-PE (Supplementary Table S2). Sequencing ladders generated by the same primer are included. (**b**) Gel shift assay of a ^32^P-labeled DNA fragment of the region upstream of *ryhB* with increasing amounts of Fur in the presence of the divalent cation MnSO_4_. Lanes 1 through 5 are Fur concentrations of 0, 100, 200, 400, and 600 nM, respectively. Lanes 6 and 7 include 600 nM of Fur incubated with probe in the presence of either 260 ng or 720 ng of non-labeled probe, respectively. (**c**) Gel shift assay of ^32^P-labeled *ryhB* probe with increasing amounts of Fur in the presence of 10 mM EDTA. Lanes 1 through 5 are Fur concentration of 0, 100, 200, 400, and 600 nM, respectively. Lane 6 includes 600 nM of Fur without EDTA, and lane 7 includes 600 nM of Fur and MnSO_4_. In figs. 2b and c, the upper bands are to be labeled with ‘Fur-probe complex’ and the lower bands with ‘Free probe’ just like in Fig. 4a.

We assessed the expression levels of *ryhB* under different iron conditions. RNA was extracted from *V. vulnificus* grown in either iron rich medium (LB broth with or without 25 μM of FeSO_4_) or under iron limiting conditions (LB broth with 200 μM of 2, 2′-dipyridyl) (Fig. 2a). In wild type MO6-24/O cells, the *ryhB* transcript is barely detectable in cells grown in LB broth with or without 25 μM of FeSO_4_, while an abundance of transcript was detected when the iron chelator 2, 2′-dipyridyl was added. In contrast, a *fur*-deletion mutant (Δ*fur*) had high amounts of the RyhB transcript regardless of iron concentrations. These results suggest that iron strongly represses the transcription of *ryhB* and that Fur is necessary for this repression.

Fur regulates gene expression in response to environmental iron conditions by binding to a specific site called the ‘Fur box’ in the promoter region of target genes^6,40,41^. To verify that Fur regulates *ryhB* expression in a similar manner, a gel-mobility shift assay was performed using a DNA fragment containing the region upstream to *ryhB* and purified Fur protein in the presence of the divalent ion Mn^2+^ (Fig. 2b). Fur specifically bound to this DNA sequence in the presence MnSO_4_ and binding was abolished if EDTA was added to sequester the divalent ion (Fig. 2c). The binding site for Fur within the *ryhB* promoter region was defined through a DNase I footprinting assay in the presence of 100 μM MnSO_4_ (Supplementary Fig. S2). The result showed that Fur protected a region from − 64 to + 6 with respect to the transcription start site, covering the consensus − 10 and − 35 promoter sequences (Supplementary Fig. S3).

### SmcR represses transcription of *ryhB* by binding to the promoter region

Our previous study showed that regulation of *vvsAB*, which encodes a biosynthetic enzyme for the siderophore vulnibactin, is coordinately regulated by both iron and quorum-sensing^6^. Furthermore, expression of the *qrr* genes, encoding small RNAs associated with quorum-sensing modulation^9^, also are regulated by iron^8^. These results point to a connection between RyhB and quorum-sensing regulation and that these are somehow associated with iron levels. In support of this prediction, analysis of the nucleotide sequences upstream of *ryhB* revealed similarities to the consensus binding motif for the quorum-sensing master regulator SmcR^42^ (Supplementary Fig. S3).

**Figure 3:**
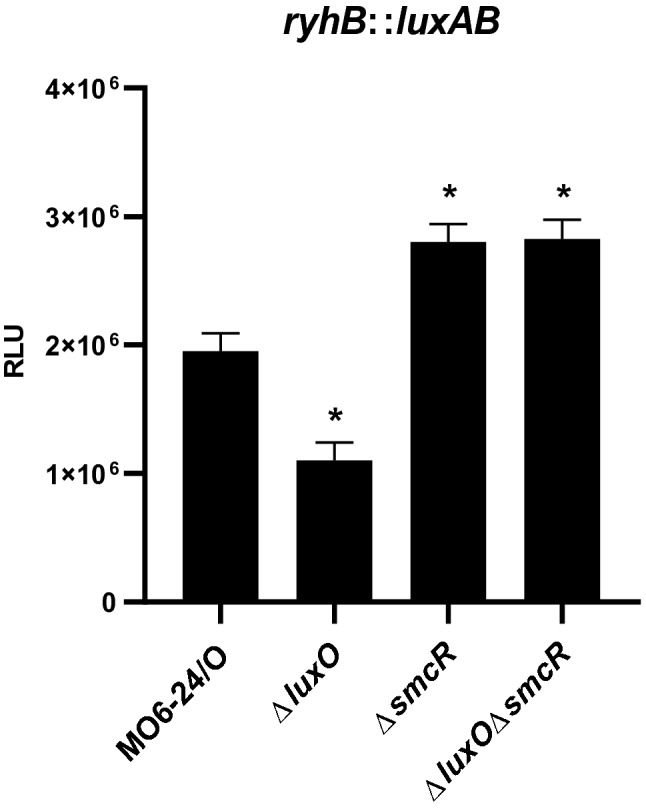
The quorum sensing master regulator SmcR represses the expression of *ryhB*. Quantitative analysis of *ryhB* transcription levels using *luxAB* as reporter genes. Luminescence activity representing the level of *ryhB* transcription was compared at early stationary phase of growth (A_600_ ≅ 1.0) for *V. vulnificus* MO6-24/O, Δ*luxO,* Δ*smcR,* and Δ*luxO*Δ*smcR* harboring pHK-*ryhB*. Relative light units (RLU) were normalized to cell density (luminescence/A_600_). Values are averages from three independent experiments, and error bars denote standard deviations. The *p*-values for comparison with MO6-24/O are indicated (Student’s *t*-test; *, 0.005 ≤ *P* < 0.05).

**Figure 4:**
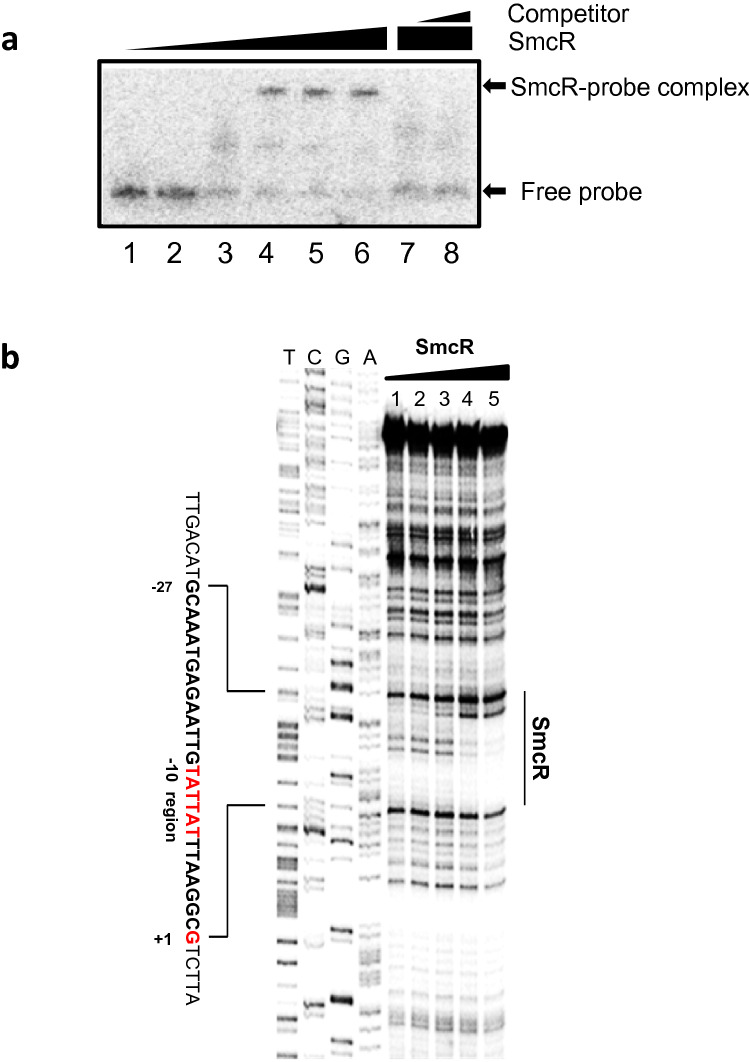
SmcR binds directly to the upstream region of *ryhB*. (**a**) Gel mobility shift assay of purified SmcR binding to a DNA fragment of the upstream region of *ryhB*. Lanes 1 to 6 represent 20 ng of ^32^P-labeled *ryhB* probe incubated with 0, 50, 100, 150, 200, and 300 nM of SmcR, respectively. Lanes 5 and 6 represent 20 ng of the labeled *ryhB* probe incubated with 250 nM of SmcR with the addition of either 260 ng or 780 ng of unlabeled probe as a competitor, respectively. (**b**) DNase I footprinting to identify the SmcR binding site in the region upstream of *ryhB*. ^32^P-labeled *ryhB* probe (100 ng) with SmcR at 0, 62.5, 125, 250, 500 and 1000 nM is included in lanes 1 to 5, respectively. Sequencing ladders were included for comparison.

To verify this, we measured the expression of *ryhB* using a *luxAB* transcriptional fusion as a reporter in the following strains: wild type MO6-24/O; a mutant with a deletion in *luxO* (Δ*luxO*) such that it lacks the cytoplasmic signal transducer that degrades SmcR at low cell density via Qrr and Hfq^8^; an *smcR*-deletion mutant (Δ*smcR*); and a *luxOsmcR* double mutant (Δ*luxO*Δ*smcR*). In both Δ*smcR* and Δ*luxO*Δ*smcR*, transcription of *ryhB* was significantly higher than that of wild type at early stationary phase (A_600_ ≅ 1.0) (Fig. 3). Conversely, in a Δ*luxO* mutant, transcription was just half that of wild type. These results indicated that SmcR represses *ryhB* expression at the transcriptional level. A DNA fragment containing the upstream region of *ryhB* was incubated with increasing amounts of purified SmcR and analyzed in a gel shift assay (Fig. 4a). As expected, SmcR bound directly to the promoter region of *ryhB*. DNase I footprinting identified the binding region as − 27 to + 1 with respect to the transcriptional start site, including a putative − 10 promoter sequence (Fig. 4b and Supplementary Fig. S3). These results indicate that SmcR represses *ryhB* transcription by directly binding to a *cis*-element upstream of *ryhB* to prevent the binding of RNA polymerase.

### RyhB promotes the production of AI-2 in *V. vulnificus*

**Figure 5:**
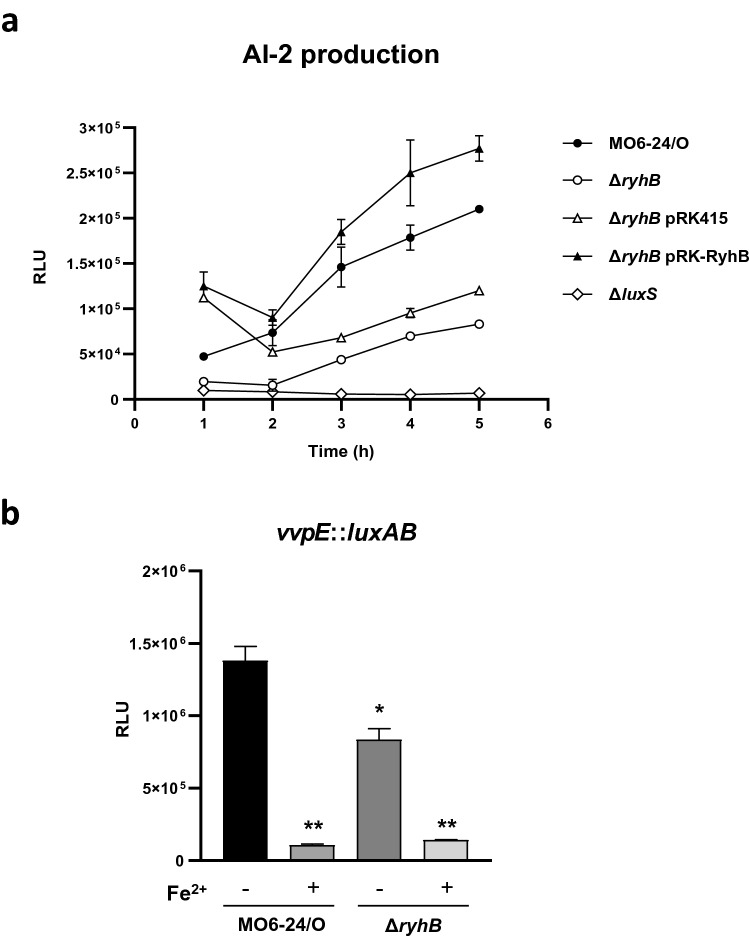
RyhB promotes AI-2 production and *vvpE* expression. (**a**) Effects of RyhB on AI-2 production were measured using the AI-2 indicator *V. harveyi* strain BB170. *V. vulnificus* strains wild type MO6-24/O, Δ*ryhB*, Δ*ryhB* with a *ryhB* clone (Δ*ryhB* pBBR12-*ryhB*), Δ*hfq*, Δ*hfq* with a *hfq* clone (Δ*hfq* pBBR12-*hfq*), and Δ*luxS* were cultured in AB minimal medium and supernatants were collected to measure AI-2 production. (**b**) Effects of RyhB on the expression of *vvpE*. *vvpE* expression was measured using a transcriptional fusion (pHK-*vvpE*) in either *V. vulnificus* wild type or the *ΔryhB* mutant. *V. vulnificus* was cultured in LBS medium supplemented with 100 μM of 2, 2′-dipyridyl to A_600_ of about 0.1. Samples were collected at late stationary phase (A_600_ ≅ 2.5) and both cell density and luminescence were measured. Relative light units (RLU) were normalized to cell density (luminescence/A_600_). Values are an average of three independent experiments and error bars denote the standard deviations. The *p*-values for comparison with MO6-24/O (without iron) are indicated (Student’s *t*-test; *, 0.005 ≤ *P* < 0.05; **, *P* < 0.005).

The results described above suggested that RyhB is involved in the iron-dependent regulation of the quorum-sensing pathway in *V. vulnificus*. To examine this further, we compared the transcription of genes related to the quorum-sensing pathway in wild-type and a *ryhB*-deletion mutant using transcriptome analysis. Expression levels of *luxPQUO* and SmcR were not significantly different in the two isotypes. However, the expression of *luxS*, encoding an AI-2 biosynthetic enzyme, was approximately 40% lower in a Δ*ryhB* mutant than in wild type (Supplementary Table S3). To verify this result, we compared both AI-2 production and LuxS expression in wild type and Δ*ryhB* mutant backgrounds. Supernatants from each of the two *V. vulnificus* strains cultured in AB minimal medium with a low iron concentration were assessed for the production of AI-2 using the bio-indicator *V. harveyi* BB170^43^ and measuring luminescence. The luminescence induced by the wild type culture supernatant was significantly higher than that of Δ*ryhB*, and introduction of an exogenous *ryhB*-expressing clone into Δ*ryhB in trans* restored luminescence to wild type levels, whereas introduction of vector alone did not (Fig. 5a). These results indicate that RyhB promotes expression of LuxS under iron-limiting conditions. We then assessed the effects of RyhB on the expression of a gene normally regulated by the AI-2 quorum-sensing system. We quantitatively measured the expression of *vvpE*, which encodes elastase, a virulence factor that is positively regulated by quorum sensing^7,44^. Iron repressed *vvpE* expression regardless of RyhB. However, in the absence of iron, expression in the Δ*ryhB* mutant was significantly lower than in wild type (Fig. 5b). This result supports the idea that enhanced production of AI-2 by RyhB promotes quorum-sensing signaling.

In summary, these results indicate that RyhB increases AI-2 mediated quorum-sensing signaling by enhancing the production of AI-2 itself, and that SmcR represses RyhB as a mechanism for feedback inhibition to repress AI-2 production. The presence of iron, as part of the Fur-iron complex, down-regulates overall quorum-sensing signaling by inhibiting the transcription of both SmcR and RyhB.

### RyhB delays the decay of *luxS* mRNA by binding directly to the 5′-UTR of LuxS

**Figure 6:**
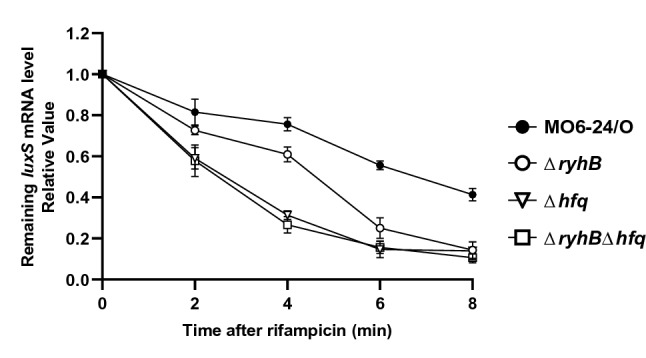
Effects of RyhB on the stability of *luxS* mRNA. Effects of RyhB and Hfq on the stability of *luxS* mRNA. The relative levels of *luxS* mRNA following rifampicin treatment were measured using qRT-PCR. *V. vulnificus* MO6, *ryhB* mutant (Δ*ryhB*), *hfq* mutant (Δ*hfq*) or *ryhB*, *hfq* double mutant (Δ*ryhB*Δ*hfq*) were cultured in AB broth for 5 h until reaching an A_600_ of 0.3. Samples were then collected at 0, 2, 4, 6, and 8 min after the addition of rifampicin (500 μg/ml), and RNA was quantified by qRT-PCR.

The next question was how RyhB enhances the production of AI-2 at the molecular level. The non-coding sRNA RyhB post-transcriptionally regulates gene expression by affecting the turnover of mRNAs in *E. coli*^26^. We assumed that RyhB would affect the *luxS* mRNA in the same manner. To test this, levels of the *luxS* transcript were measured over time after cells were treated with rifampicin. As shown in Fig. 6, *luxS* mRNA degraded faster in Δ*ryhB* than in wild type. Previous studies reported that Hfq is required for the functioning of RyhB. To test for this possibility in *V. vulnificus*, we also compared the levels of *luxS* mRNA following rifampicin treatment in wild type, *ΔryhB*, Δ*hfq*, and a *ΔryhB*Δ*hfq* double mutant. As shown in Fig. 6, in the absence of Hfq, *luxS* mRNA levels decreased significantly, independent of RyhB, indicating that even though RyhB appears to stabilize *luxS* mRNA, this function requires Hfq.

**Figure 7:**
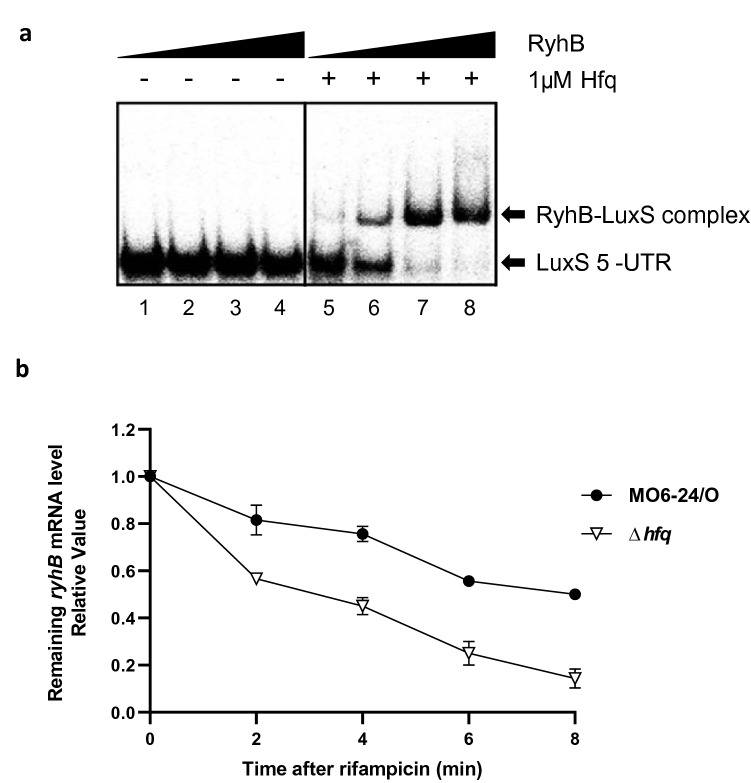
Effects of Hfq on interactions between RyhB and *luxS* mRNA. (**a**) Gel shift assay comparing the binding of RyhB to *luxS* mRNA with and without Hfq. The LuxS 5′-UTR was transcribed and labeled with ^32^P-UTP using T7 RNA polymerase. Increasing concentrations of purified RyhB were incubated with LuxS 5′-UTR with or without the addition of 1 μM of Hfq. Lanes 1 to 4 include RyhB concentrations of 0, 100, 200, and 400 nM without Hfq, respectively. Lanes 5 to 8 include RyhB concentrations of 0, 100, 200, and 400 nM with Hfq, respectively. The probes bound by RyhB are indicated with arrows. (**b**) Effects of Hfq on the stability of RyhB mRNA. Relative RyhB mRNA levels following rifampicin treatment were measured using qRT-PCR. *V. vulnificus* MO6 or the *hfq* mutant (Δ*hfq*) were cultured in AB broth for 5 h until reaching an A_600_ of 0.3. Samples were then collected at 0, 2, 4, 6, and 8 min after treatment with 500 μg/ml rifampicin for RNA purification and qRT-PCR analysis.

Generally, non-coding RNAs modulate gene expression by base pairing with target mRNAs at the 5′-UTR^45^, and the sRNA chaperone Hfq either stabilizes the sRNA or facilitates binding of the sRNA to the target^46^. We predicted that RyhB delays the decay of *luxS* mRNA by binding directly to the 5′-UTR. A ^32^P-labeled 102-bp *luxS* RNA fragment including bases − 62 to + 40 with respect to the *luxS* translation start site was expressed by in vitro transcription using T7 RNA polymerase. Full-length RyhB was also transcribed using in vitro transcription, and increasing amounts of this transcript were incubated with the labeled *luxS* mRNA probe in the presence or absence of Hfq. In the presence of Hfq, RyhB binds to the 5′-UTR of *luxS* mRNA in a concentration-dependent manner (Fig. 7a), whereas no binding was observed in the absence of Hfq. We also assessed the effect of Hfq on the half-life of RyhB and observed that stability was significantly reduced in Δ*hfq* compared to wild type (Fig. 7b). Together these indicate that RyhB binds directly to the 5′-UTR of *luxS* mRNA and stabilizes it with the assistance of Hfq.

### Confirmation of base pairing between RyhB and the 5′-UTR of *luxS* mRNA

**Figure 8:**
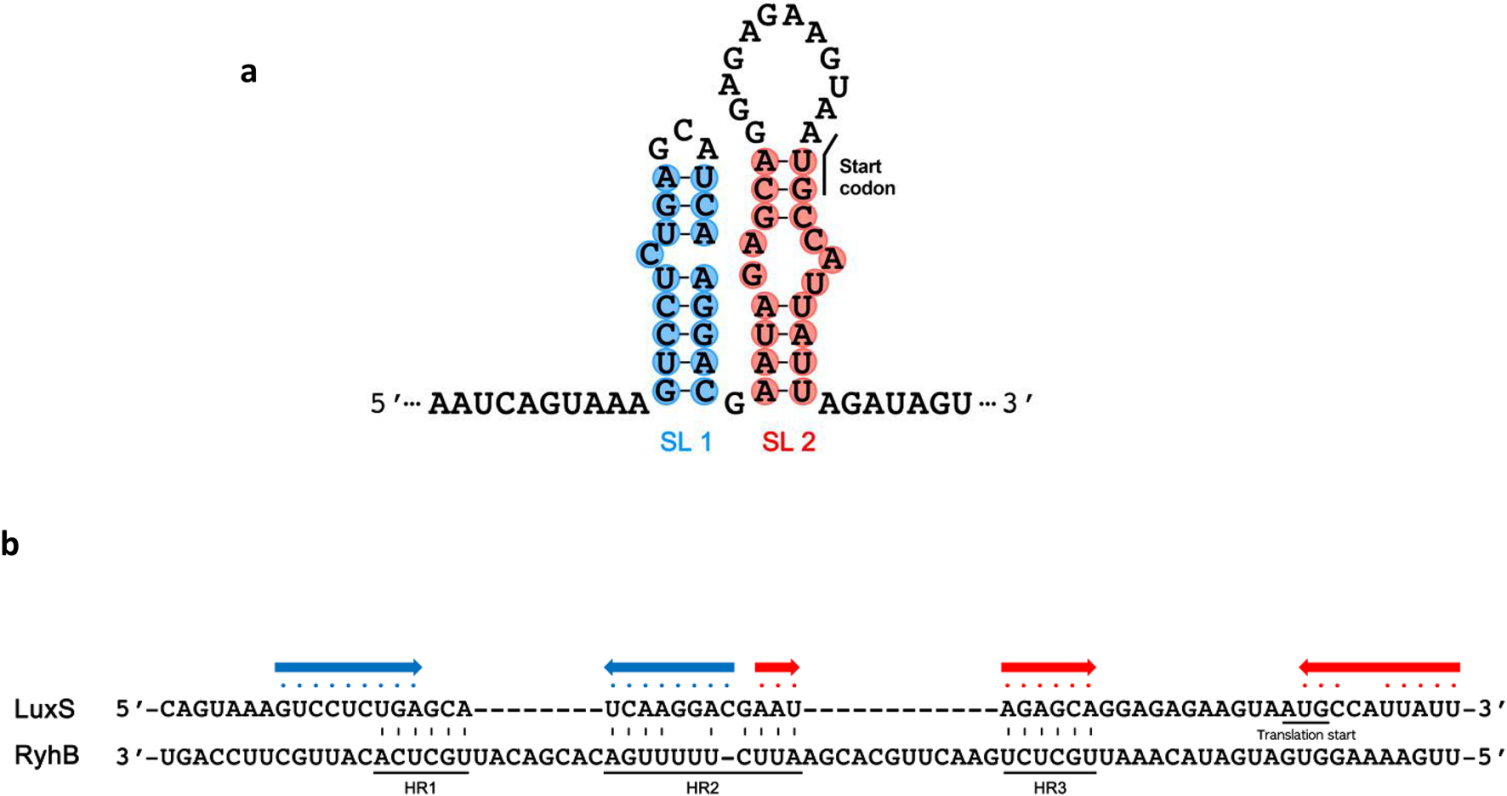
Predicted secondary structure of the 5′ end of *luxS* mRNA and potential hybridization sites between RyhB and *luxS* 5′-UTR mRNA. (**a**) The secondary structure of the 5′ end of *luxS* mRNA was predicted using mFold software. The nucleotides that potentially form stem structures are shaded in blue (SL1) or red (SL2) and the start codon is noted. (**b**) Possible regions where RyhB and the *luxS* 5′-UTR may hybridize. Three possible regions of hybridization (HR1, HR2, and HR3) are indicated and the potential stem structures from (**a**) are noted with the corresponding colored dots and arrows.

The mFold software^47^ was used to predict secondary structures that may form in the 5′-UTR of *luxS* mRNA (Fig. 8a), one of which is a stem-and-loop structure (SL2) that would obscure the start codon. Hybridization between RyhB and the 5′-UTR of *luxS* mRNA was predicted to include base pairing in three regions labeled HR (Hybridized Region) 1, 2, and 3 (Fig. 8b). If hybridization occurs at these sites, loops SL1 and SL2 would be resolved and the start codon of *luxS* mRNA would be exposed (Fig. 8b).

**Figure 9:**
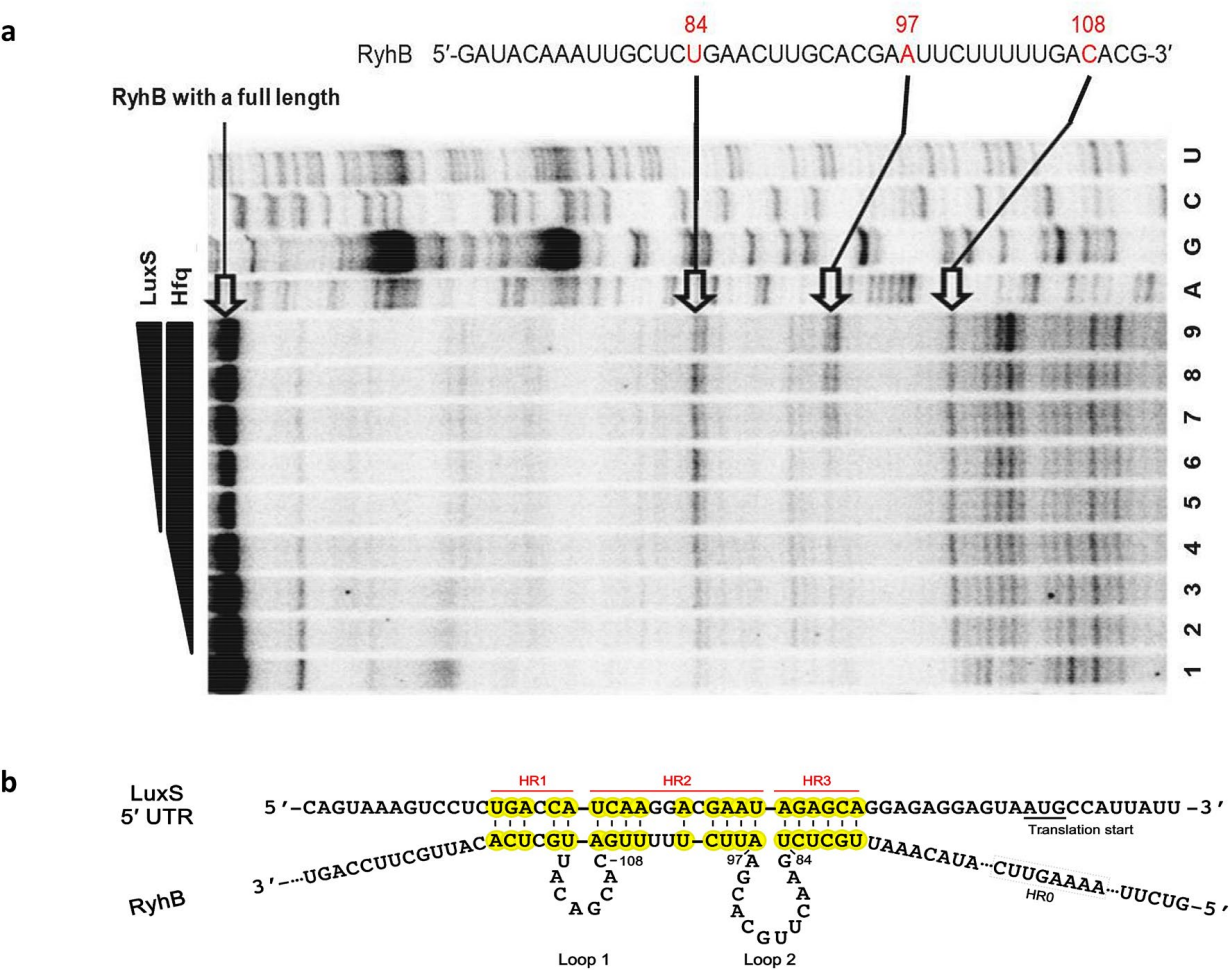
Identification of regions of RyhB RNA that hybridize with *luxS* mRNA. (**a**) Primer extension assays to determine the hybridization sites between RyhB and *luxS* RNA. Lanes 1 to 4 include Hfq at 0, 125, 250, and 500 nM, respectively. Lanes 5 to 9 include *luxS* RNA at 100, 200, 400, 600, and 800 nM incubated with 1 μM Hfq, respectively. The three distinct bands that represent higher intensities in the presence of Hfq are indicated. Base positions are numbered relative to the transcription start site of RyhB. (**b**) A model of base pairings between LuxS 5′-UTR and RyhB. Hybridization regions (HR) 1, 2, and 3 are highlighted in yellow. Loops 1 and 2 of RyhB, which form during the hybridization also are indicated. The region labeled HR0 is the site that was mutagenized to generate HR0m as a positive control.

To confirm these predictions, we performed a primer extension in the presence or absence of the 5′-UTR of *luxS* mRNA by reverse transcription using RyhB RNA as a template and primer RyhB-PE (Supplementary Table S3, Supplementary Fig. S3) which is complementary to the 3′-end of RyhB RNA. We expected that binding of RyhB to the *luxS* mRNA in the presence of Hfq would produce shorter immature cDNA products due to the formation of secondary structures between two RNA molecules that partially block the primer extension. In fact, we observed partially extended cDNA molecules that terminated at cytosine at position 108 and uracil at position 84 relative to the first base of the *ryhB* transcript (Fig. 9a,b). These two sites correspond to the 3′-end of the HR2 and HR3 regions of RyhB, respectively. cDNA terminated at adenine at position 97 also was observed. This residue is the first base at the 3′-end of Loop 2 (Fig. 9b). These results suggested that hybridization to *luxS* mRNA does indeed occur at HR2 and HR3. We speculate that there may be weak hybridization at HR1 such that termination of cDNA at this region was barely detectable. To confirm the involvement of the HR2 and 3 regions of RyhB in regulation of the *luxS* expression, we constructed derivatives of RyhB with mutations in either or both of HR2 or 3 (named HR2m, HR3m, and HR2&3m) by site-directed mutagenesis. A mutation called HR0m at a site outside of the hybridized region (HR0) was generated as a positive control (Fig. 9b and Supplementary Fig. S3).

**Figure 10:**
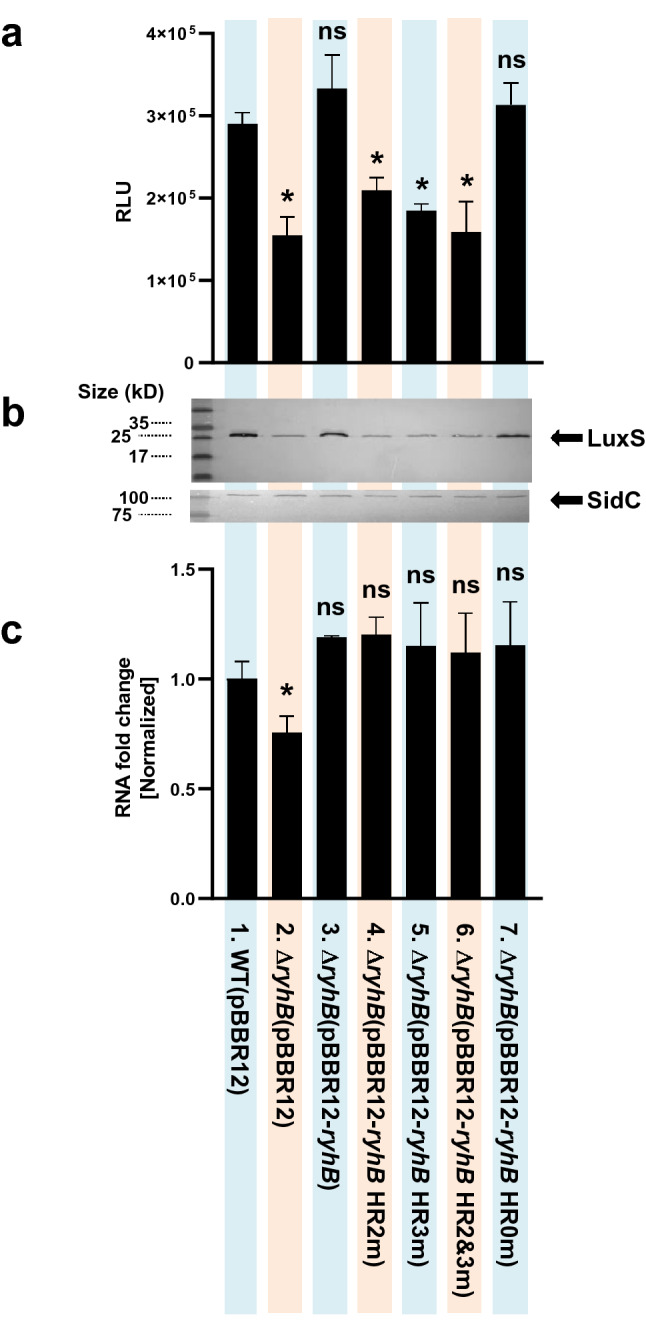
Effects of the hybridization between RhyB and *luxS* mRNA on expression of LuxS. (**a**) Influence of hybridization regions HR2 and HR3 on the production of AI-2 by LuxS as measured using the *V. harveyi* indicator strain BB170. The following *V. vulnificus* strains were cultured in AB minimal medium and sampled for the presence of AI-2 in the supernatant: 1, wild type (MO6 pBBR12); 2, *ryhB* deletion mutant (Δ*ryhB* pBBR12); 3, Δ*ryhB* with a wild type *ryhB* clone (Δ*ryhB* pBBR12-*ryhB*); 4, Δ*ryhB* with a mutated region HR2 clone (Δ*ryhB* pBBR12-*ryhB* HR2m); 5, Δ*ryhB* with mutated region HR3 clone (Δ*ryhB* pBBR12-*ryhB* HR3m); 6, Δ*ryhB* carrying a clone with both HR2 and HR3 mutated (Δ*ryhB* pBBR12-*ryhB* HR2&3m); 7, Δ*ryhB* with a clone carrying an unrelated mutated region as a control (Δ*ryhB* pBBR12-*ryhB* HR0m). The presence of AI-2 was measured in RLU, relative light units (luminescence/A_600_). Values are averages from three independent experiments and error bars denote the standard deviations. The *p*-values as compared with MO6-24/O pBBR12 are indicated (Student’s *t*-test; *, 0.005 ≤ *P* < 0.05; ns, no significant). (**b**) Effects of HR2 and HR3 on the production of LuxS as measured by western hybridization. Western blot analysis of LuxS levels in whole cell lysates was measured. As a control, insulinase enzyme (SidC) (Kim et al., 2015. *J. Biol. Chem.* 290:18708), which is not regulated by quorum-sensing, was included. (**c**) The relative mRNA levels of *luxS* were measured using qRT-PCR for each of the strains listed in 10A. Cells were cultured in AB broth for 5 h until they reached an A_600_ of 0.3, at which point samples were collected for RNA purification and qRT-PCR analysis. The *p*-values for comparison with MO6-24/O pBBR12 are indicated (Student’s *t*-test; *, 0.005 ≤ *P* < 0.05; ns, no significant).

Wild type RyhB and each of the RyhB mutations (HR0m, 2m, 3m, and 2&3m) were introduced into a *ryhB*-null mutant strain (Δ*ryhB*) and assessed for both AI-2 production using the *V. harveyi* indicator strain BB170 (Fig. 10a) and levels of LuxS by western hybridization using antibody against purified LuxS (Fig. 10b). RyhB with HR0m did not differ significantly from wild type. However, the presence of either HR2m or 3m led to significant decreases in both AI-2 production and LuxS expression, suggesting that HR2 and 3 are critical for LuxS regulation. However, the results of *luxS* qRT-PCR for wild type and the *ryhB* mutants showed that these mutations did not affect transcription levels of *luxS* under experimental conditions (Fig. 10c), suggesting that the regulation of the LuxS expression by RyhB is exerted at the translational level.

## Discussion

It is well known that an adequate concentration of iron is required for the survival of bacteria^2,48^. Nevertheless, iron is not readily available in natural environments due to its low solubility at neutral pH^49^. Furthermore, it is very difficult for pathogenic bacteria to compete with host cells for iron^50^. Therefore, pathogenic bacteria are equipped with various mechanisms to more effectively scavenge iron in the host environment. Our previous studies showed that iron levels affect quorum-sensing pathways^6,8^. This study more specifically defines the relationship between iron levels and quorum-sensing by showing that iron inhibits the production of AI-2, an initiation signal for quorum-sensing. Our observations lead us to propose that one of the most important roles for quorum-sensing is to control the timing of expression of virulence factors in pathogens as they prepare to attack host cells to acquire nutrients such as iron.

Fur is the most common regulator of iron-dependent target genes. We previously showed that cell density signals and iron converge on the AI-2 quorum-sensing pathway in *V. vulnificus*, and that the regulation is also mediated by Fur^7,8^. In the presence of iron, Fur represses the expression of major components in this pathway such as *luxO,* five *qrr* genes, and *smcR*. However*,* a deletion in *fur* did not fully abolish this repression^8^. This observation led us to search for additional factors known to be involved with iron-dependent regulation. We examined these factors and found that RyhB affects the expression of *luxS,* which encodes the biosynthesis of AI-2. Neither genetic analysis using a reporter fusion to *luxS* nor gel-shift assays using purified Fur-iron along with the upstream region of *luxS* provided any evidence that Fur directly controls *luxS* expression in *V. vulnificus* (our unpublished data). Interestingly, unlike other components of the quorum-sensing pathway, *luxS* expression appears to be controlled by RyhB at the translational level.

This mode of regulation may allow for a continuous basal level of AI-2 expression even in the presence of iron rather than the tight suppression that would occur if Fur were involved. If cells do not produce any AI-2 at all, the quorum-sensing pathway cannot quickly resume once it is shut down by the Fur-iron complex. This scenario may also explain the presence of the strong promoter sequence of *ryhB*, which is very similar to the canonical consensus promoter sequence in this organism. In this way, expression of *ryhB* is initiated swiftly once the repression exerted by Fur-iron is relieved. Employing RyhB may also provide a particular advantage in that this small RNA functions at the translational level to alter expression quickly and, due to the short half-life of the molecule, repression is rapidly relieved if the SmcR or Fur-iron signals disappear. The chaperone Hfq is absolutely required for RyhB activity. Furthermore, levels of *luxS* mRNA in a *hfq*-deletion mutant were even lower than in a *ryhB*-deletion isotype (Fig. 6), suggesting that yet other factor may exist, possibly an additional small RNA specific to *luxS*. A search for non-coding small RNAs modulated by RyhB may provide more insight into the iron-dependent modulation of genes.

Quorum-sensing signaling pathways are a valuable way for pathogenic bacteria to adapt to host environmental conditions but they are energetically expensive due to the numerous factors and variety of regulatory mechanisms that are employed. Therefore, it is necessary for cells to be equipped with the ability to control this signaling when it is not needed. For example, once a quorum-sensing signaling molecule accumulates within a closed environment, it is possible that the cell would continue to sense the signal even when expression of target genes are no longer required. The best way to avoid such a waste of energy is to lower the concentration of signaling molecules. Two possible mechanisms are either to degrade the signal molecule or to cease synthesis of the signal molecule. It is known that bacteria harbor enzymes to degrade the homoserine lactone signal molecule^51,52^. However, whether the AI-2 molecule is degraded in a similar way remains to be elucidated. To the best of our knowledge, ours is the first study to show that feedback inhibition of quorum signaling occurs through the inhibition of AI-2 synthesis. Other small RNAs called Qrrs are also known to be involved in the feedback control of quorum-sensing^8,53,54^. However, these appear to be involved in elaborate control of the signal to properly adapt to changes in cell density and not in feedback control of the quorum-sensing pathway as a whole.

AI-2 is a signal that plays a very important role in bacterial community structure, affecting inter-species communication as well as intra-species communication^9^. AI-2 signaling from pathogenic bacteria can be transmitted to other recognizable bacteria, and a decrease in AI-2 levels in response to the presence of iron can certainly affect other microbiota present in the infected regions of a host. A comparison of the gut microbiota in mice infected with wild type *V. vulnificus* and mice infected with a *luxS*-deleted isotype are significantly discernable (our unpublished data). This suggests that the presence of iron alone can indeed affect the community structure of the microbiota, in addition to influencing levels of the quorum-sensing signal molecules and related target functions. Studies on the effect of AI-2 on host microbiota in the context of iron concentrations may provide interesting insight into host-microbe interactions.

Small RNAs have been widely studied especially in the context of virulence regulation in pathogenic bacteria^31,37,38,55^. In *Vibrionacea,* small RNAs has been intensively studied in *V. cholerae*^56^. However, in other species, the role of small RNAs awaits further elucidation. The nucleotide sequences of RyhB and *luxS* mRNAs and possible hybridization between these two molecules in three related *Vibrio* spp. (*V. cholerae*, *V. parahaemolyticus*, and *V. harveyi*) demonstrated that, for all three, the start codon for *luxS* is hidden within stem-loop structures, unless binding with RyhB resolves this secondary structure (Supplementary Fig. S4), implying that this type of regulation is common among *Vibrionaceae*. Considering that major virulence factors are controlled by quorum sensing within these species, appreciation of the role of RyhB in mechanisms related to pathogenicity will provide useful information about the disease process.

Iron, or the molecules with which it interacts, may be good targets for the development of agents to control pathogenic bacteria. This study demonstrated that iron inhibits the quorum sensing signaling pathway associated with expression of virulence factors, and any tactics to enhance iron solubility may be of value to control pathogenic bacteria. Development of molecules such as anti-sense RNAs that interfere with the action of RyhB or possibly direct inhibitors of Hfq may be useful clinical approaches.

## Materials and methods

### Strains and culture condition

Strains and plasmids used in this study are listed in Supplementary Table S1. *Escherichia coli* cells were cultured in Luria–Bertani (LB) medium at 37 °C. *V. vulnificus* strains were grown at 30 °C in LB medium supplemented with 2.0% (w/v) NaCl (LBS) or in AB minimal medium (0.3 M NaCl, 0.05 M MgSO_4_, 0.2% casamino acid, 1 mM KPO_4_, 1 mM l-arginine, pH 7.5)^57^. All media components were purchased from Difco (Detroit, USA), and antibiotics were purchased from Sigma (St. Louis, USA).

### Determination of the transcription start site of *ryhB*

RNA was isolated from *V. vulnificus* using the RNase Easy Mini Kit (Qiagen, Valencia, CA), and RNA concentration was determined using a Biophotometer (Eppendorf, Hamburg, Germany). A 500 ng sample of RNA extracted from *V. vulnificus* was incubated with 5′-labeled primer RyhB-PE at 65 °C and chilled on ice. Reverse transcription was performed using a PrimeScript RT reagent kit (Takara, Tokyo, Japan). The resulting product and sequencing ladder were resolved on a 6% polyacrylamide sequencing gel to identify the transcription start site.

### Construction of *ryhB* deletions in *V. vulnificus*

A DNA fragment comprising the upstream region of *ryhB* was amplified by primers RyhB-KO-upF and RyhB-KO-upR, and ligated into a pGEM-T easy vector, generating plasmid pGEM-T-RyhBup. The downstream region of *ryhB* was amplified by primers RyhB-KO-downF and RyhB-KO-downR and ligated into a pGEM-T easy vector, generating plasmid pGEM-T-RyhBdown. Plasmid pGEM-T-RyhBup was digested with restriction enzymes *Pst*I and *Bam*HI, and the resulting DNA fragment containing the *ryhB* upstream region was ligated into plasmid pGEM-T-RyhBdown to construct pGEM-T-RyhBKO. The construction was digested with *Xho*I and *Xba*I, and subsequently ligated into pDM4 to obtain pDM4-RyhBKO which was subsequently introduced into *E. coli* S17-1 *λpir* to be mobilized into *V. vulnificus* by conjugation. Double crossover selection was performed on a 10% sucrose plate as described previously^58^. The *ryhB* deletion mutant, Δ*ryhB*, was confirmed by PCR and DNA sequencing.

### Gel shift assay

A 336-bp DNA fragment including the *ryhB* promoter region (− 213 to + 123 with respect to the transcriptional start site) was PCR-amplified using the ^32^P-labeled primers RyhB-F1 and RyhB-R1 (Supplementary Table S2). For the gel shift assay, 10 ng of labeled DNA fragment was incubated with increasing amounts of purified SmcR (0 to 1 μM) or Fur (0 to 1 μM) in a 20 μl reaction for 30 min at 37 °C. The SmcR binding reaction buffer contained 10 mM HEPES, 100 mM KCl, 200 μM EDTA, and 10% glycerol at pH 7.5. The Fur binding reaction contained 10 mM HEPES, 100 mM KCl, and 10% glycerol at pH 7.5 and was supplemented with either 100 μM MnSO_4_ or 1 mM EDTA. The binding reaction was terminated by the addition of 3 μl sucrose dye solution (0.25% bromophenol blue, 0.25% xylene cyanol, 40% sucrose) and the samples were resolved on a 6% neutral polyacrylamide gel. For gel shift assays of RyhB and LuxS 5′-UTR, 10 ng of ^32^P-labeled LuxS 5′-UTR and increasing amounts of RyhB were incubated at 70 °C for 10 min and then chilled on ice for at least 1 min. Then 2 μl of 10 × structure buffer (100 mM Tris–Cl, 50 mM magnesium acetate, 1 M ammonium chloride, 5 mM DTT, pH 7.5) and Hfq were added and incubated at 30 °C for 10 min. The binding reaction was terminated by the addition of 4 μl of sucrose dye and resolved on a 6% neutral polyacrylamide gel.

### Construction of *ryhB*::*luxAB* and *vvpE*::*luxAB* transcriptional fusions

Primers PryhB-F and PryhB-R were used for PCR amplification of the *ryhB* promoter region, which is − 251 to + 123 relative to the transcription start site. Primers PvvpE-F and PvvpE-R were used for PCR amplification of the *vvpE* promoter region. The resulting products were digested with *Kpn*I and *Xba*I (Takara, Ohtsu, Japan) and cloned into vector pHK0011 to construct pHryhB and pHvvpE, respectively, and each was conjugated into *V. vulnificus* MO6-24/O wild type, single mutants of *luxO*, *smcR*, and the *luxO*/*smcR* double mutant.

### Bioluminescence assay

Overnight cultures of *V. vulnificus* strains were washed with either LBS or AB medium and inoculated into fresh AB medium containing the appropriate antibiotic. At various growth stages, 0.006% (v/v) n-decylaldehyde was added and luminescence was measured using a luminometer (Mithras LB 940, Berthold Technologies, Bad Wildbad, Germany). Transcription levels were measured as light units normalized to cell density (relative light units: RLU) as described previously^6^.

### DNase I footprinting assay for binding of SmcR and Fur to the upstream region of *ryhB*

An end-labeled 340-bp DNA fragment consisting of bases − 217 to + 123 relative to the transcription start site of *ryhB* was generated by PCR amplification using primers ^32^P-labeled RyhB-F1 and RyhB-R1 (Supplementary Table S2). To determine the binding site of SmcR, 200 ng of the amplified DNA fragments was incubated with increasing amounts of purified SmcR or Fur for 30 min at 37 °C in 50 μl buffer (10 mM HEPES, 100 mM KCl, 200 μM EDTA, 10% glycerol, pH 7.5). To identify the Fur binding region, 200 ng of the resulting DNA fragments was incubated for 30 min with increasing amount of purified Fur in 50 μl of binding solution (10 mM HEPES, 100 mM KCl, 10% glycerol, 100 μM MnSO_4_, pH 7.5). After supplementing 50 μl of CaCl_2_-MgCl_2_ solution (5 mM CaCl_2_, 10 mM MgCl_2_) to the binding reaction, 0.25 unit of DNase I (Promega, Madison, USA) was added and allowed to react for 1 min at 37 °C before terminating with 90 μl stop solution (200 mM NaCl, 30 mM EDTA, 1% SDS). To precipitate the DNA, 500 μl ethanol was added and samples were incubated on ice for 30 min, after which pellets were collected, washed with 70% ethanol, and suspended in 10 μl loading buffer (98% formamide, 0.1% xylene cyanol, 0.1% bromophenol blue). The resulting product and sequencing ladder generated with the ^32^P-labled RyhB-F1 primer (Supplementary Table S2) were resolved on a 6% polyacrylamide sequencing gel.

### Detection of AI-2 production

The AI-2 assay was performed using the *V. harveyi* reporter strain BB170 as previously described^43^. Briefly, BB170 was prepared by culturing overnight in LB broth at 30 °C, then washing twice and diluting 1:3000 in fresh AB minimal medium. Test cultures of *V. vulnificus* were grown overnight in LBS broth and then washed once before diluting 1:250 in fresh AB broth. Each hour, A_600_ was measured and 10 μl of cell-free supernatant was collected and mixed with 90 μl of diluted BB170, prepared as described above, in 96 well plates at 30 °C. The luminescence was measured using a Mithras LB 940 multimode microplate reader (Berthold, Germany).

### RNA synthesis by in vitro transcription

Template DNA of *ryhB* and *luxS* was prepared for in vitro transcription by PCR using primers containing the T7 promoter sequences as shown in Supplementary Table S2. RNA was synthesized by in vitro transcription using T7 RNA polymerase (Takara, Tokyo, Japan) using the prepared template DNA at 37 °C following the protocol provided and was further purified using a Monarch RNA Cleanup Kit (NEB, Cambridge, USA).

### Determination of the regions of RyhB RNA and *luxS* mRNA that hybridize with each other using primer extension with reverse transcriptase

RyhB (50 ng) was incubated with LuxS 5′-UTR and ^32^P-labeled primer RyhB-PE, which is complementary to RyhB from residues 167 and 189 relative to the transcription start site of *ryhB*, at 65 °C for 10 min and then chilled on ice. Hfq and 10 × structure buffer (100 mM Tris–Cl, 50 mM magnesium acetate, 1 M ammonium chloride, 5 mM DTT, pH 7.5) were added and the mixture was incubated at 30 °C for 10 min. Lastly, dNTPs and 1 unit of SuperScript III reverse transcriptase (Invitrogen, Carsbad, USA) were added and incubated at 30 °C for 1 h. Reactions were terminated at 85 °C for 5 min, and samples were resolved on a 6% polyacrylamide sequencing gel alongside a sequencing ladder generated by the same primer.

### Purification of LuxS and Hfq proteins

A DNA fragment covering the 519-bp *luxS* open reading frame (ORF) encoding the 172-amino acids of LuxS was PCR-amplified using primers STREP-LUXSF and STREP-LUXSR (Supplementary Table S2). The amplified fragment was sub-cloned into pASK-IBA-7 (IBA, Göttingen, Germany) which generates a fusion between the Strep-tag and the N-terminus of the expression protein. The resulting Strep-tagged *luxS* construct was transformed into *E. coli* BL21 (DE3) (Novagen, Madison, USA), and over-expressed by inducing with 10 μg/ml anhydrotetracycline. The bacterial pellets were suspended in W buffer (100 mM Tris–Cl, 150 mM NaCl, pH 8.0), sonicated, and then centrifuged at 13,000 rpm for 15 min. The resulting supernatant was applied to 1 ml of Strep-Tactin-Sepharose resin (IBA, Göttingen, Germany) and bound proteins were eluted with E buffer (100 mM Tris–Cl, 150 mM NaCl, 1 mM EDTA, 2.5 mM desthiobiotin, pH 8.0). The eluted proteins were assessed for purity using 15% sodium dodecyl sulfate–polyacrylamide gel electrophoresis (SDS-PAGE). To purify the Hfq protein, DNA spanning the 261-bp Hfq ORF encoding 86 amino acids of Hfq was PCR-amplified using primers STREP-HFQF and STREP-HFQR (Supplementary Table S2). The amplified fragment was cloned into pASK-IBA-7 to generate pASK-IBA-Hfq (Supplementary Table S1), and Hfq was purified as described above.

### Construction of a *ryhB*-deletion and series of site-directed mutations in *ryhB*

A DNA fragment containing the complete sequence of *ryhB* was PCR-amplified using primers CryhB-F and CryhB-R (Supplementary Table S2). The resulting product was ligated into pGEM-T easy vector (Promega). After confirmation by sequencing, the DNA fragment containing the *ryhB* sequence was cut with *Kpn*I and *Xba*I and cloned into pRK415, generating pRK-RyhB. Nucleotides that potentially bind to LuxS 5′-UTR were mutated using the EZchange Site-directed Mutagenesis Kit (Enzynomics, Deajeon, Korea). Primers ryhB_SDM2_F and ryhB_SDM2_R were used to construct pRK-RyhB2m; primers ryhB_SDM3_F and ryhB_SDM3_R were used to construct pRK-RyhB3m; all four primers (ryhB_SDM2_F&R, ryhB_SDM3_F&R) were used to construct pRK-RyhB2&3m; and primers ryhB_SDM_CON_F and ryhB_SDM_CON_R were used to construct the control pRK-RyhB0m (Supplementary Table S2). The resulting constructs were mobilized from S17-1 to mutant strain Δ*ryhB*. Conjugants were selected in thiosulfate-citrate-bile salts-sucrose agar (TCBS) plate medium supplemented with 1 μg/ml tetracycline.

### Preparation of polyclonal rabbit antibody against purified LuxS and western hybridization

Purified LuxS was used to produce polyclonal rabbit antibodies (Ab Frontier, Seoul, South Korea). For LuxS expression western blot analysis, *V. vulnificus* MO6-24/O wild type or mutants were cultured in LBS broth for 6 h, then diluted 1:100 in fresh LB broth. After 3 h of growth, cells were collected and washed twice with PBS, after which 20 μg of each lysate was resolved by SDS-PAGE and transferred to Hybond P membrane (Amersham, Arlington Heights, USA). The membrane was incubated first with polyclonal rabbit antibodies against LuxS (1:2000) and then with goat anti-rabbit IgG-AP (1:5000) (Santa Cruz Biotechnology, CA, USA). LuxS expression was visualized using Western blotting luminal reagent (Santa Cruz Biotechnology, CA, USA).

## Supplementary Information

Supplementary Information.
