# Supplementary material for: Quorum sensing and iron-dependent coordinated control of autoinducer-2 production via small RNA RyhB in Vibrio vulnificus
Source: Sci Rep. 2022 Jan 17;12:831. doi: 10.1038/s41598-021-04757-9 (PMC8764119; doi:10.1038/s41598-021-04757-9)
Supplement: Supplementary file 1 — Supplementary Information. [file 41598_2021_4757_MOESM1_ESM.pdf]

# SUPPLEMENTARY MATERIAL

# Quorum Sensing and Iron-Dependent Coordinated Control of Autoinducer-2 Production via Small RNA RyhB in *Vibrio vulnificus*

**Keun-Woo Lee[1], Yancheng Wen[1, 2], Na-Young Park[1], Kun-Soo Kim[1*]**

**Table S1. Strains or plasmids used in this study.**

| Strains or plasmids | Derivation / relevant characteristics | Reference or source |
|---|---|---|
| **Strains** | | |
| ***E.coli*** | | |
| DH5α | λ⁻ φ80d*lacZ* Δ*M15* Δ(*lacZYA- argF*)U169 *recA1 endA1 hsdR17* ($r_K^-m_K^-$) *supE44 thi-1 gyrA relA1* | Our Collection |
| S17-1 | [C600::RP4-2 (Tc::Mu)(Km::Tn7) *thi pro hsdRM*$^+$ *recA*, $Tp^r$ | 1 |
| S17-1 *λpir* | S17-1 with *λpir* lysogen | 1 |
| BL21(DE3) | $F^-$ *ompT hsdSB* ($r_B^-m_B^-$) *gal dcm* (DE3) | Novagen |
| ***V. vulnificus*** | | |
| MO6-24/O | Pathogenic clinical isolate | 2 |
| Δ*ryhB* | Derivative of MO6-24/O with a deletion in *ryhB* | This study |
| Δ*fur* | Derivative of MO6-24/O with a deletion in *fur* | This study |
| Δ*luxS* | Derivative of MO6-24/O with a deletion in *luxS* | This study |
| **Plasmids** | | |
| pASK-IBA-Hfq | pASK-IBA7 with *V. vulnificus hfq* | This study |
| pHryhB | pHK0011 with *ryhB* promoter fused to *luxAB* | This study |
| pRK-RyhB | pRK415 containing *V. vulnificus ryhB* | This study |
| pRK-RyhB2m | pRK-RyhB with a mutation at HR-2 of RyhB | This study |
| pRK-RyhB3m | pRK-RyhB with a mutation at HR-3 of RyhB | This study |
| pRK-RyhB2&3m | pRK-RyhB with a mutation at HR-2 and 3 of RyhB | This study |
| pRK-RyhB0m | pRK-RyhB with a mutation at upstream of RyhB as a negative control | This study |

## REFERENCES

1. Simon R, Priefer U, Pühler A. 1983. A broad host range mobilization system for *in vivo* genetic engineering: transposon mutagenesis in gram negative bacteria. Nat Biotechnol 1: 784-791.

2. Reddy GP, Hayat U, Abeygunawardana C, Fox C, Wright AC, Maneval DR, Bush CA, Morris JG. 1992. Purification and determination of the structure of capsular polysaccharide of *V. vulnificus* MO6-24. J Bacteriol 174(8):2620-30. doi: 10.1128/jb.174.8.2620-2630.1992.

3. Milton DL, O'Toole R, Horstedt P, Wolf-Watz H. 1996. Flagellin A is essential for the virulence of *Vibrio anguillarum.* J Bacteriol 178(5):1310-9. doi: 10.1128/jb.178.5.1310-1319.1996.

4. Jeong HS, Jeong KC, Choi HK, Park KJ, Lee KH, Rhee JH, Choi SH. 2001. Differential expression of *Vibrio vulnificus* elastase gene in a growth phase-dependent manner by two different types of promoters. J Biol Chem 276(17):13875-80. doi: 10.1074/jbc.M010567200.

## SUPPLEMENTAL MATERIAL

**Table S2. Primers used in this study**

| Name | Nucleotide sequence (5′ to 3′) |
|---|---|
| **Construction of *ryhB*, *luxS*, and *fur* mutant** | |
| RyhB-KO-upF | CTCGAGCATACCTCGATGTTGTACGC |
| RyhB-KO-upR | CCAATAAGCCTTAATAATAACAATTCT |
| RyhB-KO-downF | TTAAGGCTTATTGGTTTTTTTCTGTAC |
| RyhB-KO-downR | TCTAGAACCACCGCAATACGATCAGA |
| LuxS-KO-upF | CTCGAGATACCTATTTGGCCGATTACTT |
| LuxS-KO-upR | GGATCCTCTCCTGCTCTATTCGTCCTT |
| LuxS-KO-downF | GGATCCATTGGTGTAAAGTCGGGCTAA |
| LuxS-KO-downR | GCATGCATATGCTGGTTTCCATTTTGG |
| Δfur_FF_xbaI | TCTAGAGACTTCTTCTCGATATTG |
| Δfur_FR_speI | ACTAGTAGCATCCTTTAGCGCTTG |
| Δfur_BF_speI | ACTAGTGACGCACATAAACGTAAG |
| Δfur_BR_xhoI | CTCGAGTAACCATCCAAATAAGCC |
| **Primer extension** | |
| RyhB-F1 | TTTACGTTTTGTCAGGAAGATAA |
| RyhB-R1 | TGTGAGCAATGTCGTGTCA |
| RyhB-PE | TGCGGTGAAGCTAATTCGTTGAA |
| LuxS-PE+116 | CGTAAGTCAAACACGGTAATGGT |
| **Transcriptional fusion construction** | |
| PryhB-F | GGTACCTTGCGAGGTTATGAGGCTG |
| PryhB-R | TCTAGATGTGAGCAATGTCGTGTCA |
| **Protein expression** | |
| STREP_LUXSF | CCGGAATTCATGCCATTATTAGATAG |
| STREP_LUXSR | CCCTCGAGTTAATCCACTTTGAGCT |
| STREP_HFQF | CCGGAATTCATGGCTAAGGGGCAATC |
| STREP_HFQR | CCCTCGAGTTAATCTTCCGATTTCTC |

**Table S2. Primers used in this study (continued)**

| Name | Nucleotide sequence (5′ to 3′) |
|---|---|
| ***In vitro* transcription template** | |
| T7-RyhB-F | TAATACGACTCACTATAGGGGTCTTAGGGAATGAGGAAAAG TTCA |
| T7-RyhB-R | AAAAAGCGACATCGTTTGATGTCGCTAATACGACTCACTAT AGGGTGGTTTTTCT |
| T7-LuxSUTR-F | AAATCAGTAAAGTCCTCTG |
| T7-LuxSUTR-R | TGCGAGTGTGGTCAACGGTAAAACT |
| **Construction of a *ryhB*-deletion and series of mutated *ryhB*** | |
| CryhB-F | CCCTCTAGACGTCTTAGGGAATGAGGAAA |
| CryhB-R | GGGGTACCTGGACAAAGATAATCACTGAGAAA |
| ryhB_SDM_CON_F | TCTTAGGGAATGAGGGGGGACCTACAAGGATGTTGATTTA |
| ryhB_SDM_CON_R | TAAATCAACATCCTTGTAGGTCCCCCCTCATTCCCTAAGA |
| ryhB_SDM2_F | ACTTGCACGACGGAGGGTTTCCACGACATTGC |
| ryhB_SDM2_R | GCAATGTCGTGGAAACCCTCCGTCGTGCAAGT |
| ryhB_SDM3_F | AAAGGTGATGATACAAATGTGGAGGAACTTGCACGAATTC |
| ryhB_SDM3_R | GAATTCGTGCAAGTTCCTCCACATTTGTATCATCACCTTT |
| **Real time PCR** | |
| luxS-qPCR-F | AGCACCTGTACGCAGCATTTAT |
| luxS-qPCR-R | CAGATGCCACGTCTTGCTCACT |
| **Gel shift assay** | |
| ryhB_upstream_F | AAATGGCTCATAAAGCTTG |
| ryhB_upstream_R | TCCTTGTGAACTTTTCCT |

*Nucleotides modified for the generation of restriction site or for site-directed mutagenesis are underlined.

**Table S3. RNA sequencing with *V. vulnificus* MO6-24/O and Δ*ryhB*.**

| GenBank annotation number | Gene name | Product | Expression fold (Δ*ryhB*/WT) |
|---|---|---|---|
| VVMO6_04125 | *luxP* | Autoinducer-2 binding periplasmic protein | 0.86 |
| VVMO6_04124 | *luxQ* | Autoinducer-2 sensor kinase/phosphatase | 0.81 |
| VVMO6_02022 | *luxU* | Phosphorelay protein | 1.17 |
| VVMO6_02023 | *luxO* | Regulatory protein | 0.98 |
| VVMO6_00513 | *luxS* | Autoinducer-2 production protein | 0.62 |
| VVMO6_00535 | *smcR* | LuxR-type quorum-sensing regulator | 0.85 |

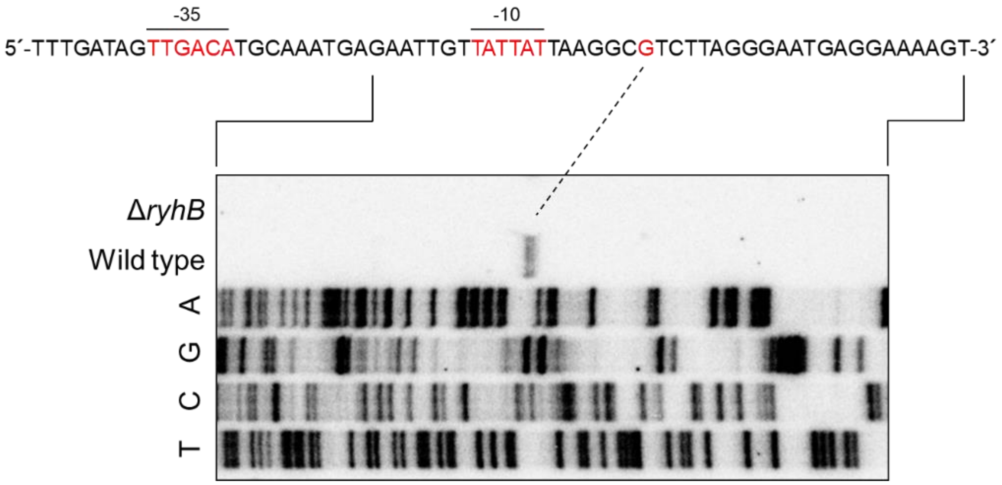


**Figure S1 . Identification of the transcription start site of RyhB**

The transcription start site of *ryhB*. Primer extension reactions were carried out on RNA extracted from either *V. vulnificus* wild type or a *ryhB* deletion mutant, both of which were cultured in LB broth with 200 µM of 2, 2′-dipyridyl. The transcription start site from wild type cells is indicated and was absent in the *ryhB* mutant, as predicted. The sequencing ladder from the wild type strain is included and putative -10 and -35 sequences are noted.

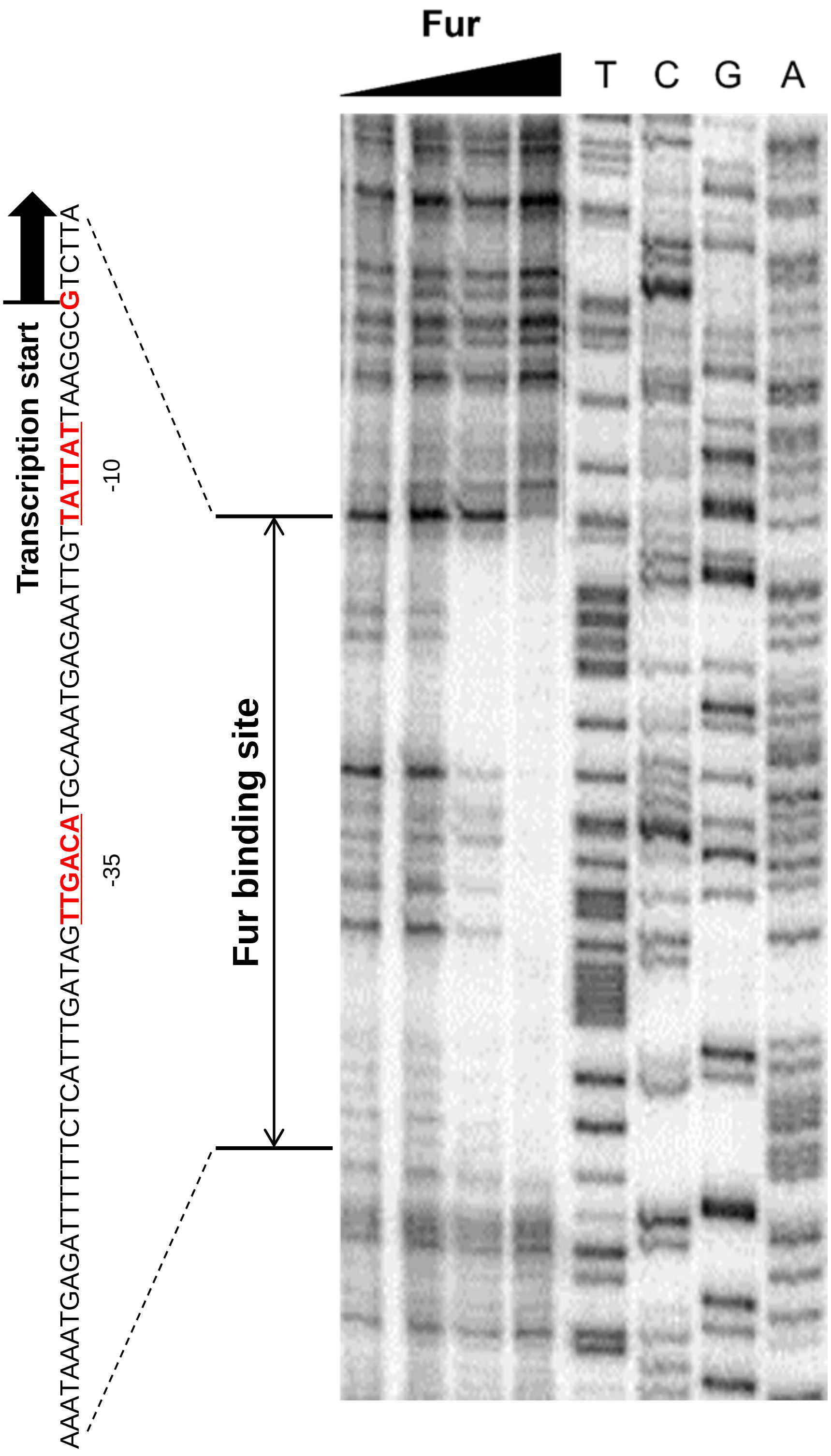


**Figure S2. Fur binding site in the upstream region of *ryhB*.**
DNaseI protection of the *ryhB* promoter region by the Fur-iron complex. 100 ng of $^{32}$P-labeled *ryhB* probe was included in each lane, and lanes 1 to 5 represent Fur concentrations of 0, 62.5, 125, 250, 500, and 1000 nM, respectively. A sequencing ladder is included for comparison. The region protected by Fur (-64 to +6) is indicated and numbered with respect to the RyhB transcription start site.

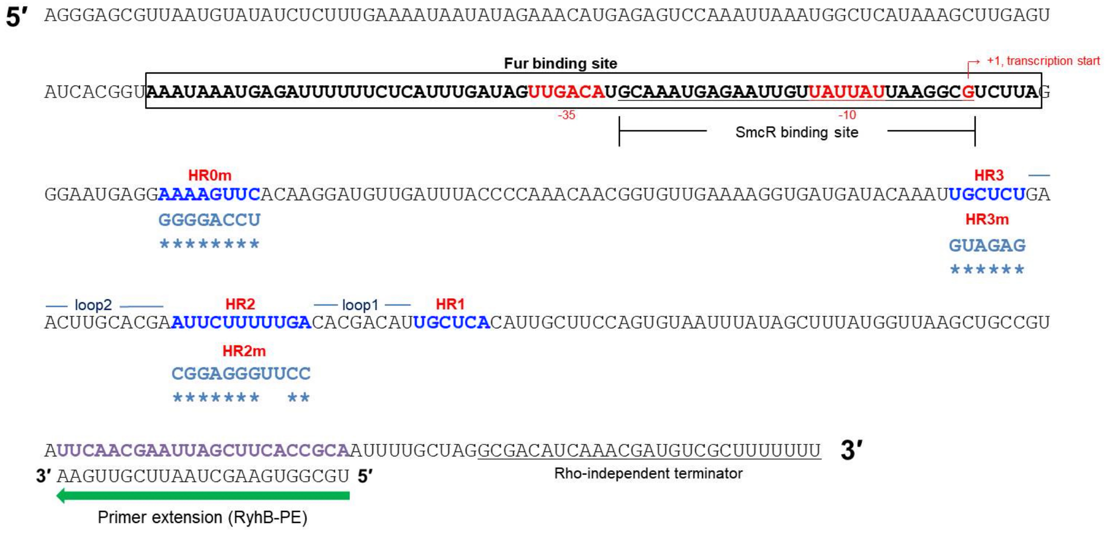


**Figure S3. Genetic map of RyhB.**

The -35 and -10 promoter consensus sequences and a *rho*-independent terminator are indicated. The Fur binding site spanning -64 to +6 is boxed. The SmcR binding site at -27 to +1 is also indicated. Loops 1 and 2, labeled above each sequence, are regions that form loop structures when hybridized with the 5′-UTR of LuxS. HR1, 2, and 3, which are regions that hybridize with *luxS* mRNA, are indicated with bold blue letters and are labeled in red. Nucleotide sequences in pale blue text shown below each of the HR regions indicate the bases that were mutagenized in this study. The sequence of primer RyhB-PE used for the primer extension experiments presented in Figure 9a is indicated with a green arrow.

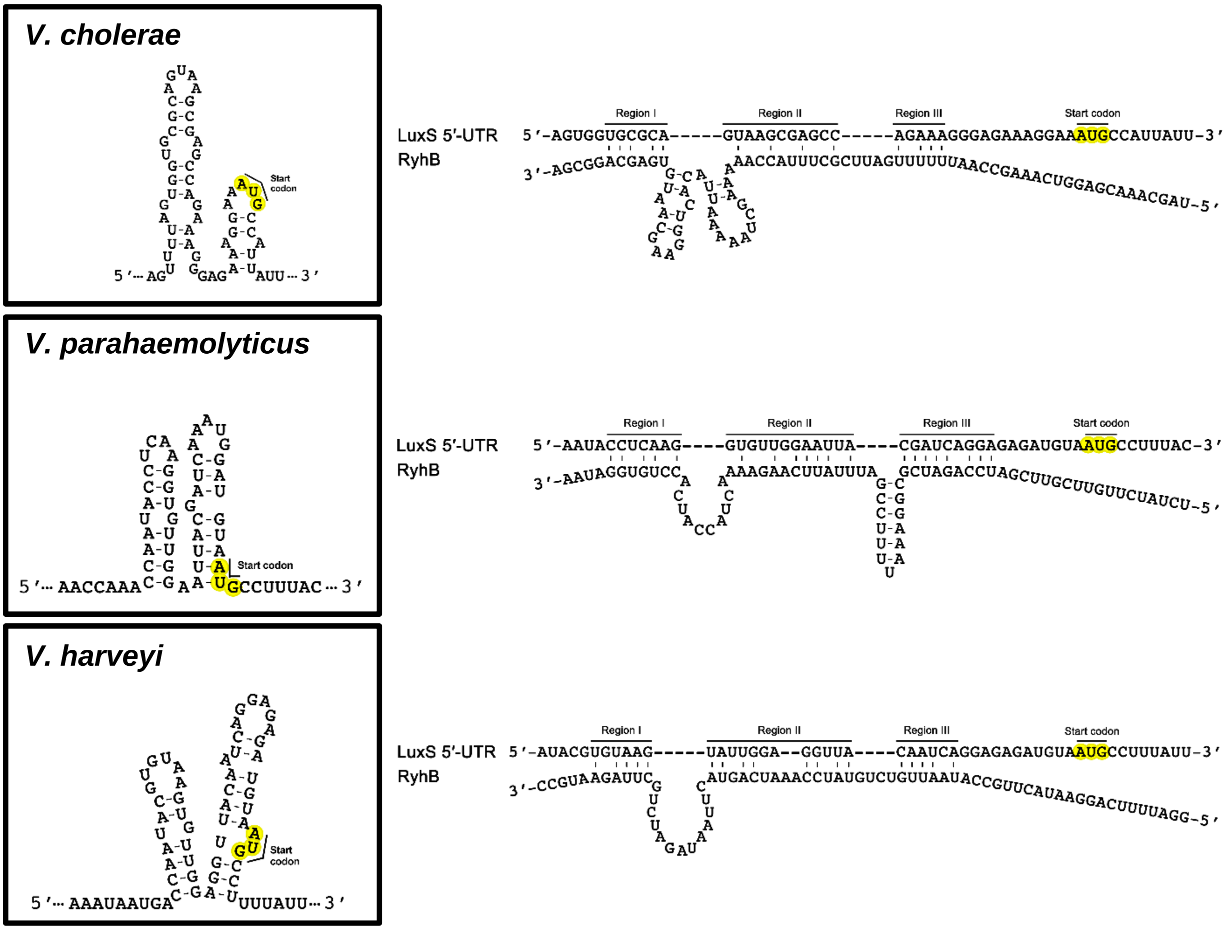


**Figure S4. A comparison of RyhB sequences between related *Vibrio* spp.** The secondary structures of the 5′-end of *luxS* mRNA from *Vibrio cholerae*, *Vibrio parahaemolyticus*, and *Vibrio harveyi* were predicted using the web software (https://rna.urmc.rochester.edu/RNAstructureWeb/Servers/Fold/Fold.html). Predicted sites of hybridization between each LuxS 5′-UTR and the respective *ryhB* RNA sequences are shown in the right panels and numbered relative to the start codon of LuxS.

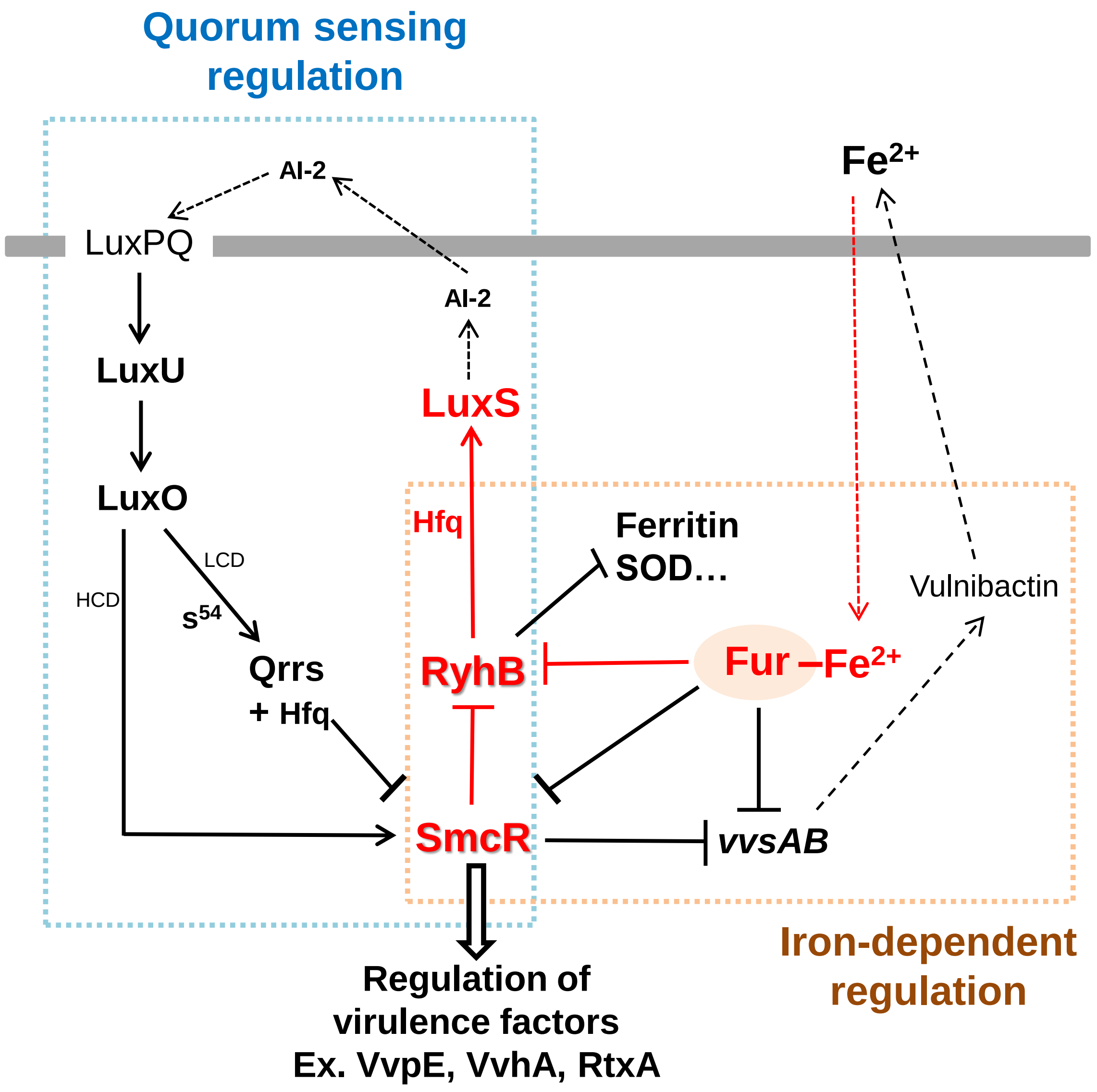


**Figure S5. A model of quorum-sensing and Fur gene regulation in *Vibrio vulnificus*.** A model showing inter-connections between quorum-sensing signaling and iron-dependent regulation that lead to the coordinated control of virulence factors in response to both cell density and iron. Novel findings from this work are indicated in red.

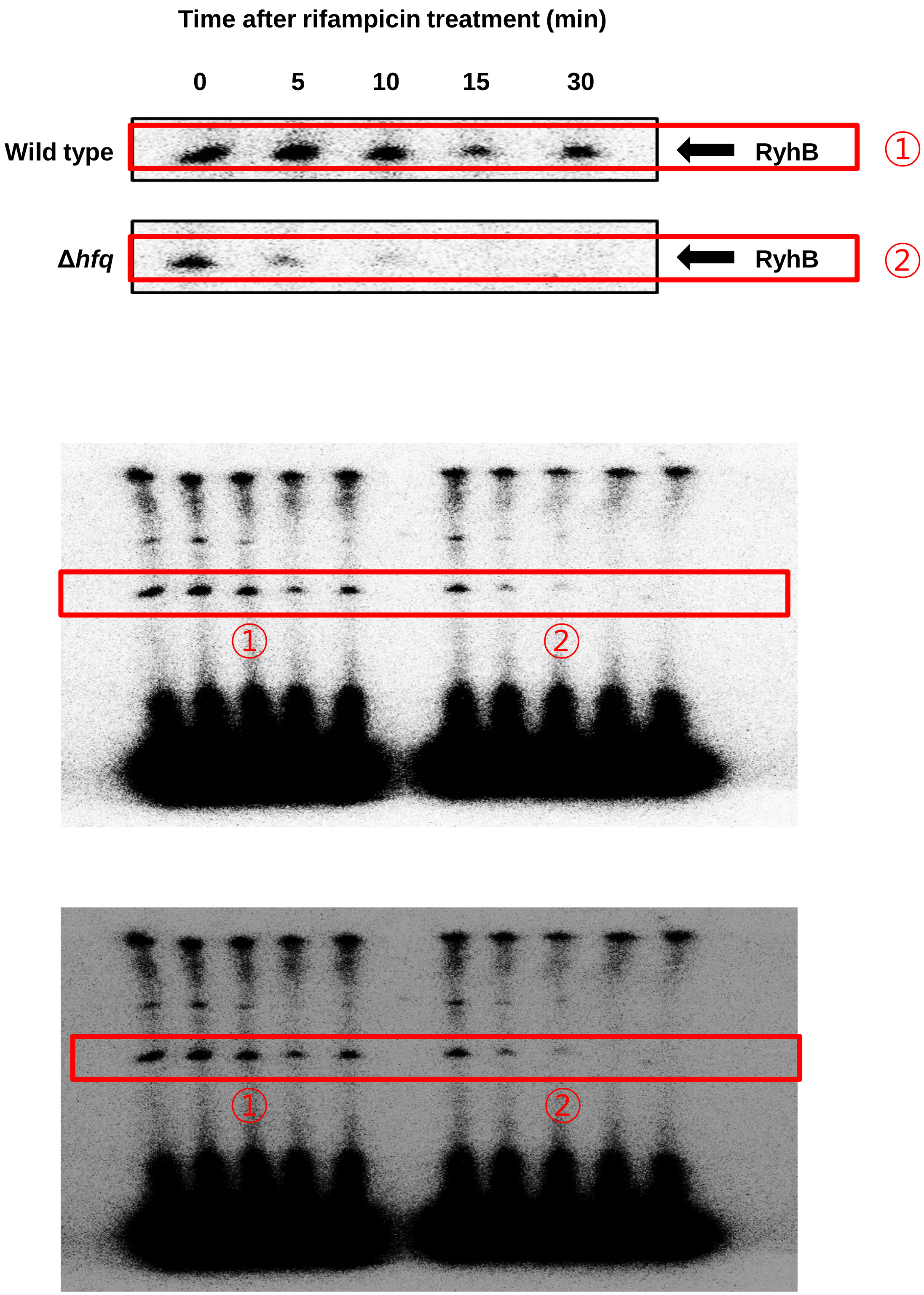


**Figure S6. Original images of Figure 1a at different exposures.**

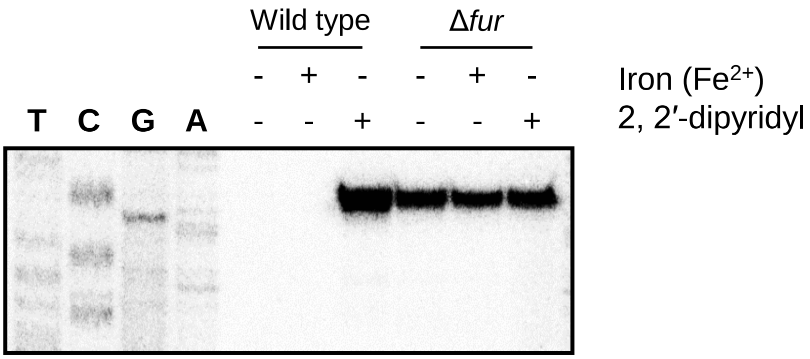


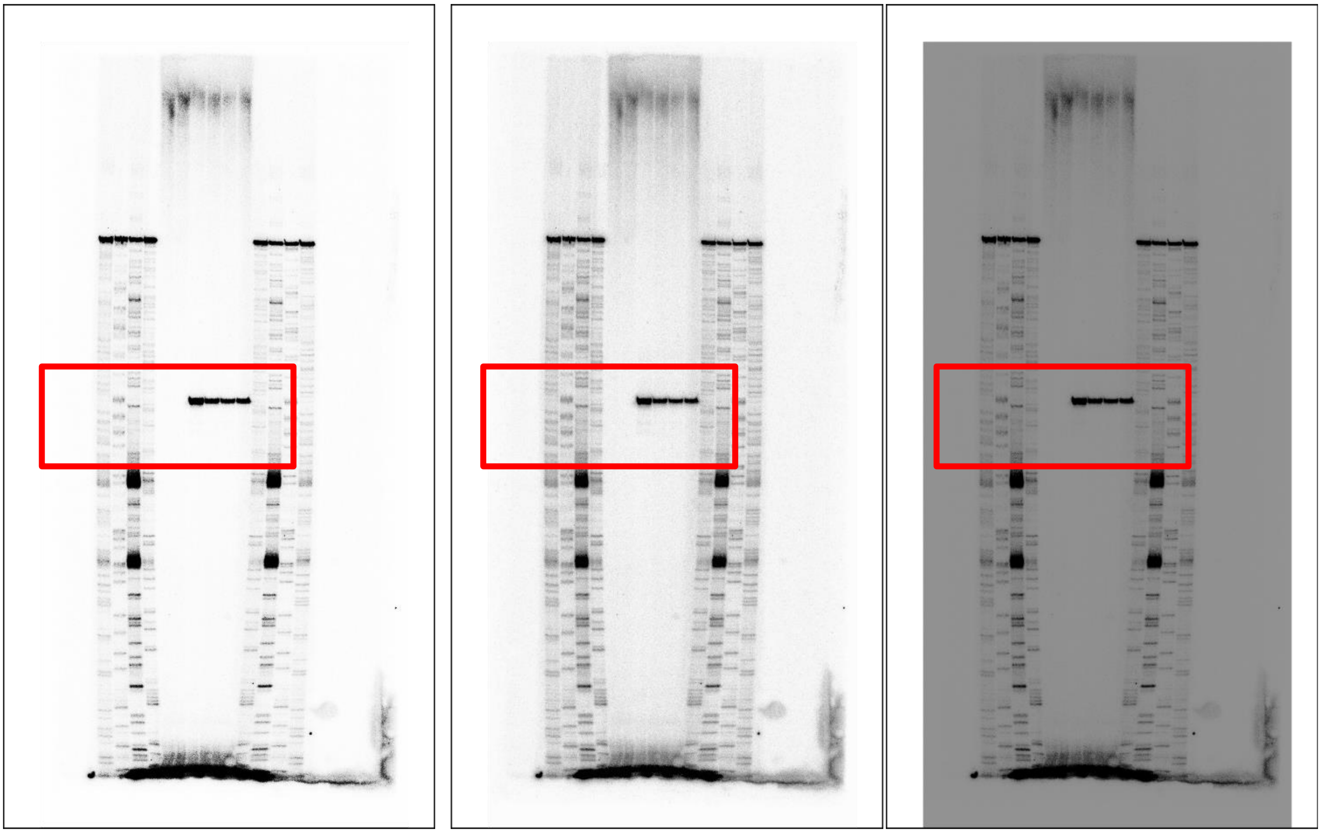

**Figure S7. Original images of Figure 2a at different exposures.**

**Figure 2b**

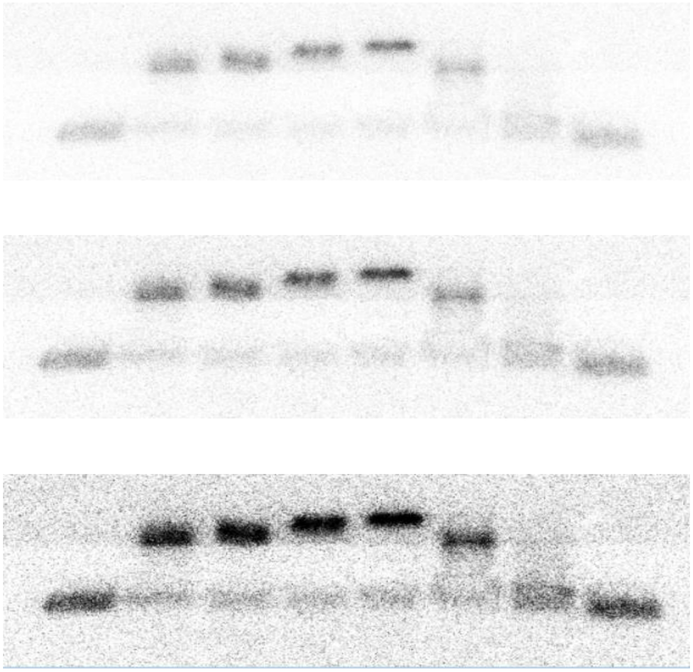

**Figure 2c**

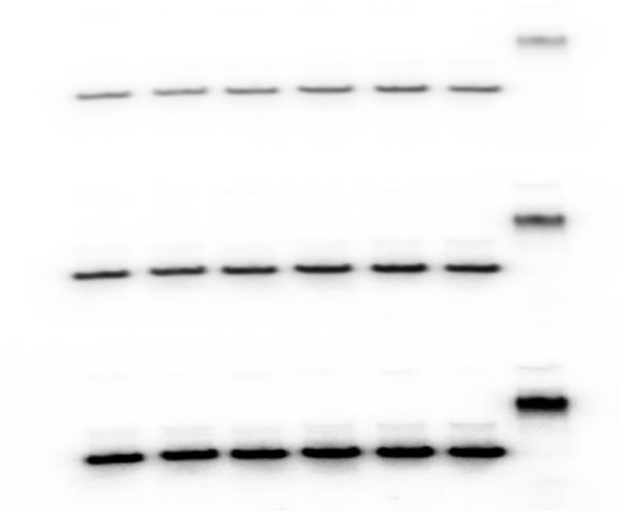

**Figure S8. Original images of Figure 2b and c at three different exposures.**

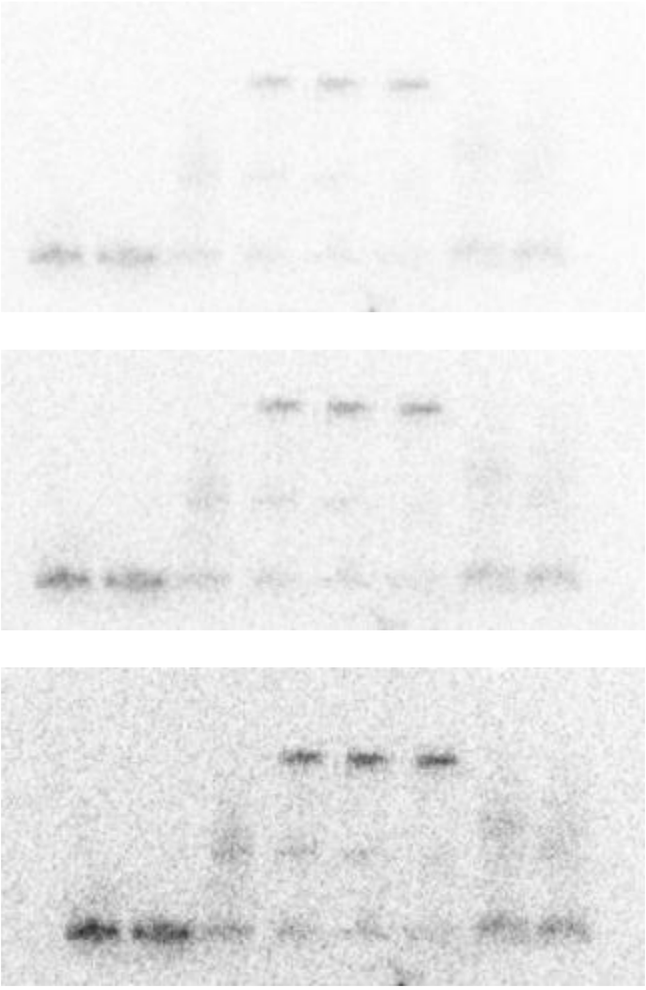

**Figure S9. Original images of Figure 4a at different exposures.**

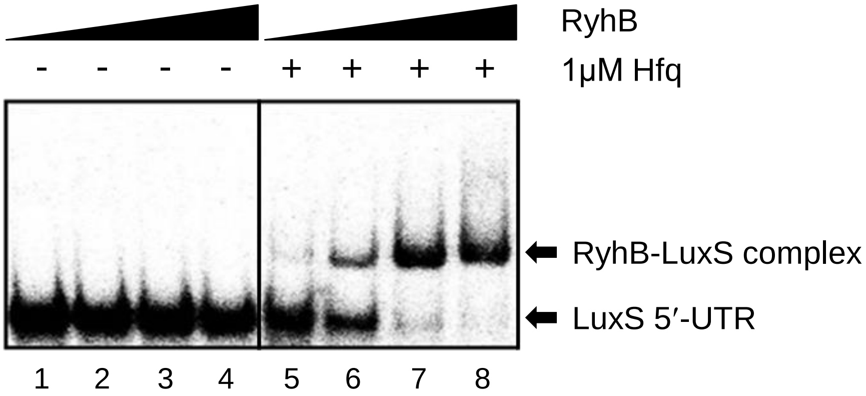


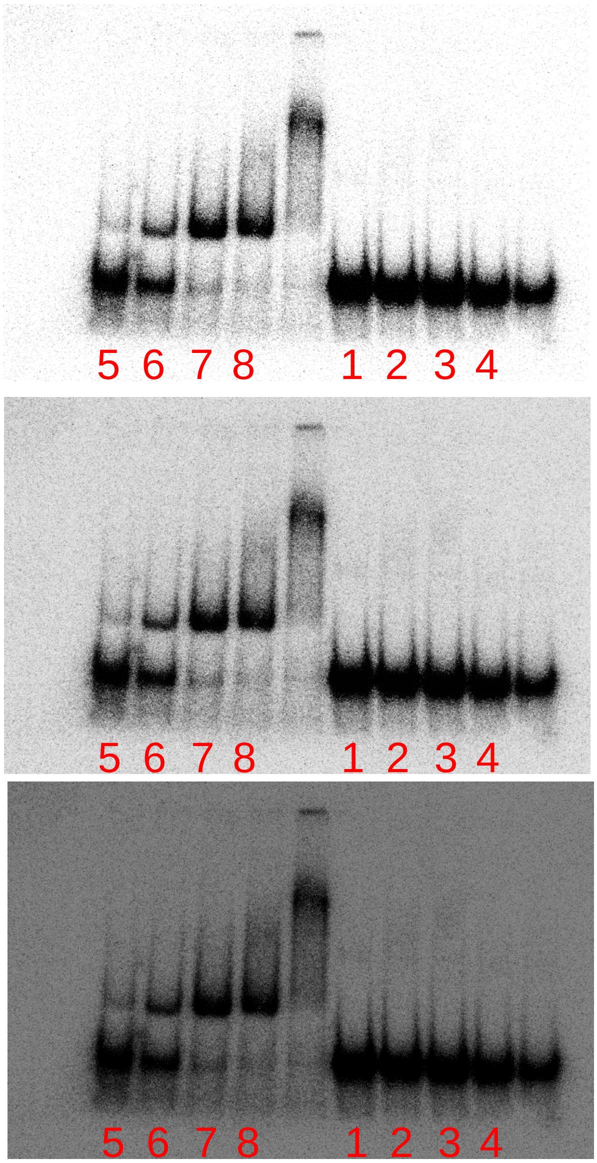


**Figure S10. Original images of Figure 7a at different exposures.**

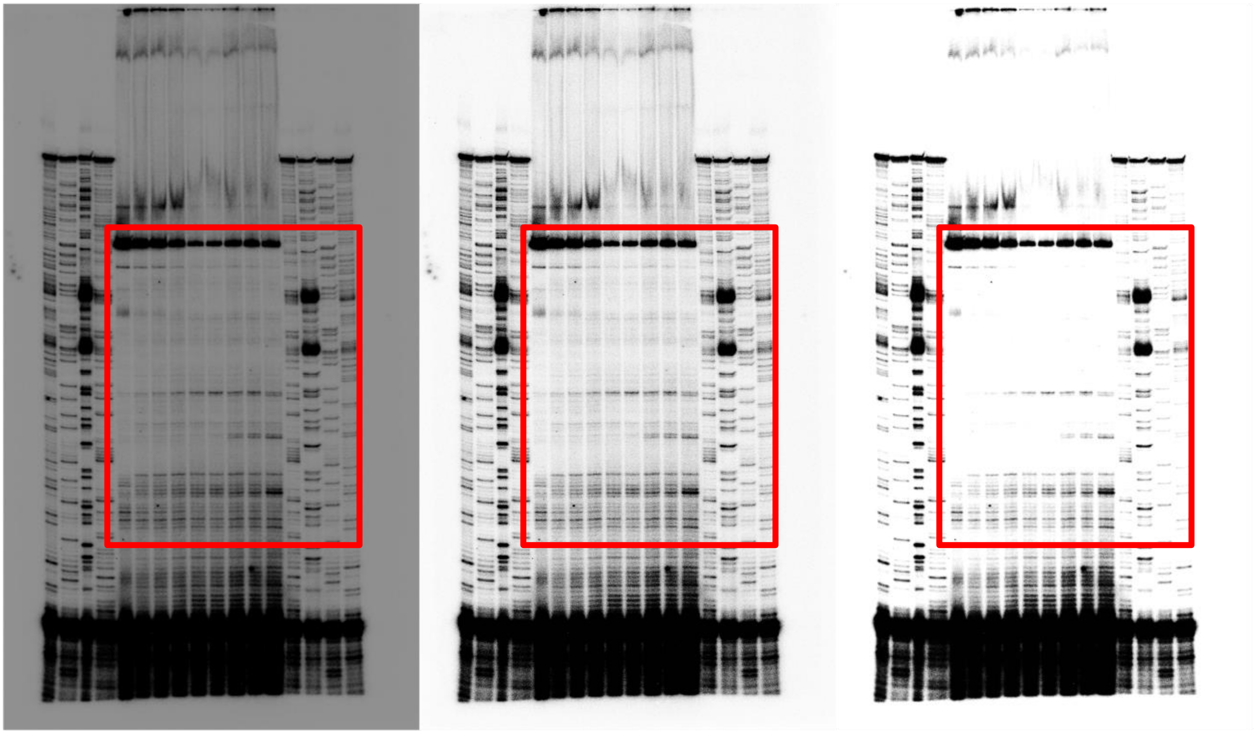

**Figure S11. Original images of Figure 9a at different exposures.**

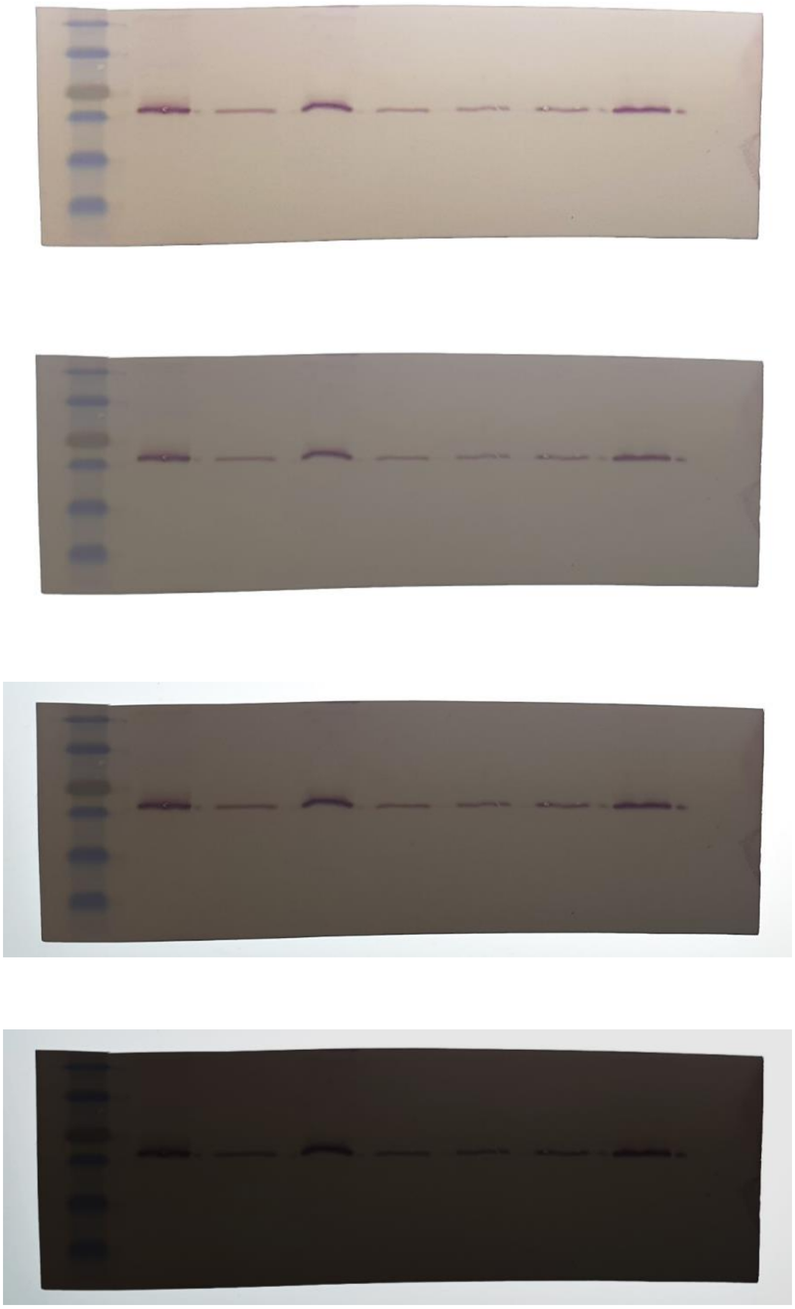

**Figure S12. Original images of Figure 10b at different exposures.**
